# Syntheses of Novel 4-Substituted *N*-(5-amino-1*H*-1,2,4-triazol-3-yl)pyridine-3-sulfonamide Derivatives with Potential Antifungal Activity

**DOI:** 10.3390/molecules22111926

**Published:** 2017-11-07

**Authors:** Krzysztof Szafrański, Jarosław Sławiński, Anna Kędzia, Ewa Kwapisz

**Affiliations:** 1 Department of Organic Chemistry, Medical University of Gdańsk, Al. Gen. J. Hallera 107, 80-416 Gdańsk, Poland; 2 Department of Oral Microbiology, Medical University of Gdańsk, ul. Dębowa 25., 80-204 Gdańsk, Poland; anak@gumed.edu.pl (A.K.), kwapisz@gumed.edu.pl (E.K.)

**Keywords:** sulfonamides, pyridine-3-sulfonamides, 1,2,4-triazole, antifungal agents, *Candida albicans*, anticancer screening

## Abstract

Candidiasis represent a serious threat for patients with altered immune responses. Therefore, we have undertaken the synthesis of compounds comprising a pyridine-3-sulfonamide scaffold and known antifungally active 1,2,4-triazole substituents. Thus a series of novel 4-substituted *N*-(5-amino-1*H*-1,2,4-triazol-3-yl)pyridine-3-sulfonamides have been synthesized by multistep reactions starting from 4-chloropyridine-3-sulfonamide via *N*′-cyano-*N*-[(4-substitutedpyridin-3-yl)sulfonyl]carbamimidothioates which were further converted with hydrazine hydrate to the corresponding 1,2,4-triazole derivatives **26**–**36**. The final compounds were evaluated for antifungal activity against strains of the genera *Candida*, *Geotrichum*, *Rhodotorula*, and *Saccharomycess* isolated from patients with mycosis. Many of them show greater efficacy than fluconazole, mostly towards *Candida albicans* and *Rhodotorula mucilaginosa* species, with MIC values ≤ 25 µg/mL. A docking study of the most active compounds **26**, **34** and **35** was performed showing the potential mode of binding to *Candida albicans* lanosterol 14α-demethylase. Also in vitro cytotoxicity of selected compounds have been evaluated on the NCI-60 cell line panel.

## 1. Introduction

The arylsulfonamides, derived from the antibacterial drug sulfanilamide, constitute an important class of biologically active compounds with a broad spectrum of pharmacological applications. Versatile biological properties of sulfonamides include antibacterial [1,2], antidiabetic [3], diuretic [4], antiglaucoma [5], antiviral [6,7], anti-inflammatory [8,9] or anticancer activity [10,11,12]. Our long-term studies on pyridine-3-sulfonamides derivatives were focused on the synthesis of 4-substituded pyridine-3-sulfonamides with both primary and secondary sulfonamide moieties, and exploration of their multidirectional biological activity: inhibition of carbonic anhydrase isozymes [13,14,15], in vitro anticancer properties [15,16] and antibacterial activity [15]. Due to the fact that mycoses are becoming a growing public health problem, mostly for patients with altered immune function as a consequence of premature birth, organ transplantation, primary immune deficiency, HIV/AIDS or cancer chemotherapy [17,18], we intended to extend our research to antifungal activity. Therefore we have synthesized a series of novel *N*-(5-amino-1*H*-1,2,4-triazol-3-yl)pyridine-3-sulfonamide derivatives and then evaluated them against yeast and yeast like species. Triazole derivatives (commonly named “azoles” together with imidazole derivatives) usually show an antifungal molecular mechanism of action based on inhibition of the cytochrome P-450-dependent lanosterol 14α-demethylase (CYP51) [19] and are the most commonly used group of antifungal drugs [20,21]. Due to its great importance, the topic of new antifungal derivatives is still widely explored [22,23,24,25]. Actually great attention is being paid to the synthesis of new hybrid compounds, among which the newly presented derivatives can also be included, that involve two different pharmacophores, one of which is a 1,2,4-triazole ring [26,27,28]. Furthermore, along with the development of organic synthesis techniques, new methods allowing to direct modification of side chain of 1,2,4-triazole scaffold have emerged [29,30], providing a tool to modify the existing lead structures.

## 2. Results and Discussion

### 2.1. Chemistry

**Scheme 1:**
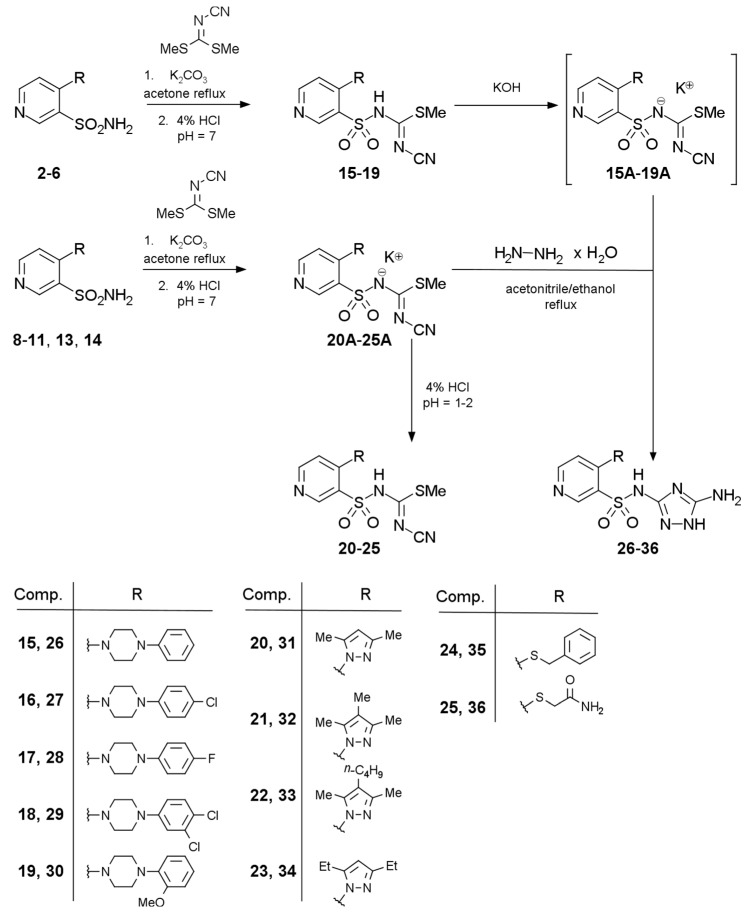
Synthesis of 4-substituted *N*-(5-amino-1*H*-1,2,4-triazol-3-yl)-pyridine-3-sulfonamides **26**–**36**.

The general synthetic routes for the preparation of the desired *N*-(5-amino-1*H*-1,2,4-triazol-3-yl)pyridine-3-sulfonamides **26**–**36** are shown in Scheme 1.

The necessary starting materials, i.e. 4-substituted pyridine-3-sulfonamides **2**–**6**, **8**–**11** and **13**–**14** were obtained according to the previously described methods [13,15], (see Scheme S1 in Supplementary Materials). Thus, target *N*-(5-amino-1*H*-1,2,4-triazol-3-yl)pyridine-3-sulfonamides **26**–**36** were synthesized in convenient two-step reactions [31] by treatment of primary pyridine-3-sulfonamides **2**–**6**, **8**–**11** and **13**–**14** with dimethyl *N*-cyanoiminodithiocarbonate in boiling acetone in the presence of anhydrous potassium carbonate, and then in the next stage of the reaction sequence, with 99% hydrazine hydrate in acetonitrile or ethanol at reflux (Scheme 1).

Facile isolation of *N*′-cyano-*N*-[(4-Subst.-pyridin-3-yl)sulfonyl]carbamimidothioate derivatives **15**–**19** in which the 4-pyridine substituent is an electron donating 4-arylpiperazine moiety, consisted of acidification of the initially formed potassium salts with 4% hydrochloric acid to pH 7, giving the desired pure product (Scheme 1). On the other hand, in the case of compounds containing an electron withdrawing substituent, such as 1*H*-pyrazol-1-yl (compounds **8**–**11**) or alkylthio groups (compounds **13** and **14**), we found that the corresponding potassium salts of type **20A**–**25A** were stable and amenable to direct isolation when acidified to pH 7, while isolation of protonated compounds **20**–**25** required stronger acidification to pH 1–2 with 4% hydrochloric acid. In the next step, the previously obtained methyl *N*′-cyano-*N*-[(pyridin-3-yl)sulfonyl]carbamimidothioates **15**–**25** in the form of the corresponding potassium salts **15A**–**25A**, in reaction with 99% hydrazine hydrate in boiling acetonitrile (compounds **26**–**30**) or ethanol (compounds **31**–**36**) furnished the *N*-(5-amino-1*H*-1,2,4-triazol-3-yl)pyridine-3-sulfonamide derivatives **26**–**36** in moderate to good yields of 40–69% (Scheme 1).

**Scheme 2:**
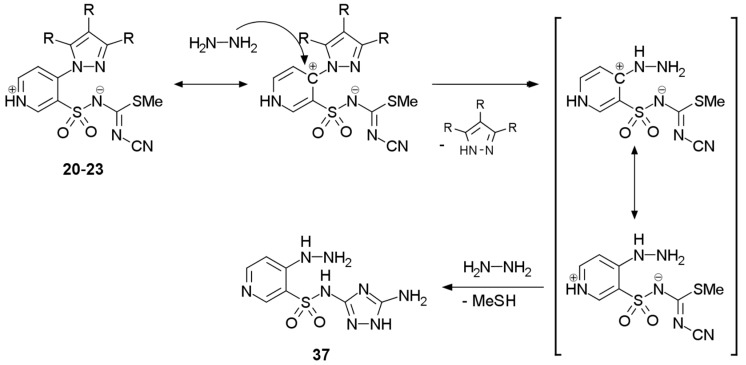
Proposed mechanism of the formation of undesirable product: *N*-(5-amino-1*H*-1,2,4-triazol-3-yl)-4-hydrazinopyridine-3-sulfonamide (**37**).

Interestingly, it has been found, that treatment of protonated form of methyl *N*′-cyano-*N*-[(pyridin-3-yl)sulfonyl]carbamimidothioates **20**–**23** with 99% hydrazine hydrate led to the formation of only compound **37** having the hydrazine moiety in position 4 instead of a pyrazol-1-yl ring (Scheme 2).

The proposed explanation of the observed reaction pattern (Scheme 2) can be justified by the known fact that compounds with the 1-alkyl-2-cyano-3-(pyridine-3-sulfonyl)guanidine structure adopt a zwitterionic form [32]; therefore, similar methyl *N*′-cyano-*N*-[(pyridin-3-yl)sulfonyl]-carbamimidothioate derivatives **20**–**23** that also show strong acidic properties should be present in the form of inner salts. Intramolecular protonation of the pyridine nitrogen atom facilitates the attack of the nitrogen nucleophile at position 4, which leads to the substitution of the pyrazole ring by hydrazine. In the final step the strong acidic *N*-cyanoisothiourea moiety is cyclized to the 1,2,4-triazole ring.

All the synthesized compounds were characterized by IR and ^1^H-NMR spectroscopy and elemental analyses (C, H, N). In the IR spectra of the methyl *N′*-cyano-*N*-[(pyridin-3-yl)sulfonyl]carbamimidothioates **15**–**25** the characteristic absorption bands corresponding to the stretching vibration of cyanide (C≡N) group appeared in the range of 2161–2178 cm^−1^ and disappeared in the final *N*-amino-1*H*-1,2,4-triazol derivatives **26**–**37** after cyclization. Similarly the ^1^H-NMR spectra of compounds **15**–**25** show singlets at 2.15–2.38 ppm corresponding to the SCH_3_ group which do not occur in the ^1^H-NMR spectra of the triazole derivatives. The strongly acidic NH proton of compounds **15**–**19** occurs as very broad signal with a chemical shift over 13.6 ppm. Surprisingly the corrresponding NH proton signal of compounds **20**–**25** is not observed at 14 ppm, however a substantial water signal usually occurring in DMSO-*d*_6_ at 3.35 ppm is found downfield as a broad signal at about 6 ppm, probably due to fast exchange with the acidic NH proton. Compounds **26**–**37** however are characterized by broad amino NH_2_ group signals in the range of 5.82–6.05 ppm.

### 2.2. Antifungal Activity 

Compounds **26**–**36** were evaluated for their in vitro antifungal activity on 31 yeast strains belonging to the genera of *Candida*, *Geotrichum, Rhodotorula*, and *Saccharomycess* isolated from patients with candidiasis of the oral cavity and respiratory tract (*Candida albicans*—eight strains, *Candida glabrata*—four strains, *Candida guilliermondii*—two strains, *Candida krusei*—three strains, *Candida lusitaniae*—two strains, *Candida parapsilosis*—three strains, *Candida tropicalis*—three strains, *Candida utilis*—one strain, *Geotrichum candidum*—two strains, *Rhodotorula mucilaginosa*—two strains, *Saccharomyces cerevisiae*—one strain). Also six standardized strains were used in the test: *Candida albicans* ATCC 10231, *Candida glabrata* ATCC 66032, *Candida krusei* ATCC 14243, *Candida lusitaniae* ATCC 34499, *Candida parapsilosis* ATCC 22019 and *Candida tropicalis* ATCC 750. The minimal inhibitory concentration (MIC), defined as concentration of tested compound which causes complete growth inhibition, was determined by means of the dilution technique in the Sabouraud’s agar (Table 1). Fluconazole was chosen as reference drug.

Among tested yeast-like species, *Candida albicans* turned out to be the most sensitive to the tested derivatives, showing susceptibility to eight compounds (**26**, **28**, **30**–**32** and **34**–**36**) with MIC values lower or comparable than the reference drug (MIC = 25–100 μg/mL). A good activity profile against *Rhodotorula mucilaginosa* was also observed for compounds **26**, **28**, **32**, **35**, and **36**, which exhibited greater efficacy than fluconazole (MIC ≥ 100 μg/mL). Furthermore, several compounds showed outstanding activity towards some yeast strains, i.e., compound **36** against *Candida glabrata* (MIC ≥ 25 μg/mL), **26** against *Candida guilliermondii* (MIC ≥ 12.5 μg/mL), **32** and **35** against *Candida krusei* (MIC ≥ 25 μg/mL and MIC ≥ 12.5 μg/mL) and **31** against *Candida tropicalis* (MIC ≥ 25 μg/mL). Lack of sensitivity to the test compounds was however observed for strains of *Candida parapsilosis*, *Geotrichum candidum* and *Saccharomyces cerevisiae*.

The structure-activity analysis revealed that among the 4-(4-phenylpiperazin-1-yl)pyridine-3-sulfonamide derivatives **26**–**30** compound **26**, with an unsubstituted benzene ring **26** or compound **28** substituted with fluorine atom are some of the most active compounds. On the other hand, compounds containing one or two chlorine atoms in the phenylpiperazine substituent like **27** and **29** didn’t demonstrate any antifungal activity. Similarly in the series of 1*H*-pyrazole derivatives **31**–**34**, lack of a substituent (compounds **31**, **34**) or the presence of a methyl group (compound **32**) at the position 4 of the pyrazole ring exerted a positive effect on antifungal properties, while the long aliphatic *n*-butyl chain in compound **33** resulted in loss of activity.

### 2.3. In Silico Docking Studies

To assess the possible ability of the new compounds to bind to lanosterol 14α-demethylase (CYP51), which is molecular target for clinically used azole-antifungals, molecular docking studies were performed. For these studies compounds **26, 34** and **35** were chosen, due to their highest overall activity and varied types of substituents in position 4 of the pyridine ring (*N*-phenylpiperazine in **26**, pyrazole in **34** and an alkylthio moiety in **35**). Also fluconazole, and posaconazole, drugs known to inhibit CYP51, were taken as the positive control.

Because *Candida albicans* turned out to be the most sensitive from among the tested species docking of the compounds was performed in the crystallographic structure of *C. albicans* lanosterol 14α-demethylase [33] (PDB ID:5FSA) co-crystallized with posaconazole, obtained from the Protein Data Bank website. The docking simulations into the active site of the enzyme was performed using the Autodock Vina software [34].

First, in order to validate the docking methodology, we performed a redocking of posaconazole, the former co-crystallized ligand with CYP51, in its active site. The root mean square deviation (RMSD) value between the top ranked predicted conformation and the observed X-ray crystal structure of ligand, was 1.198 Å (a value below 2 Å point indicates that the docking protocol was validated).

The docking parameters obtained for the best scored conformations shows that investigated compounds display more favorable estimated binding free energy, −9.9 kcal/mol for compound **26**, −9.3 kcal/mol for compound **35** and −9.1 kcal/mol for compound **34**, respectively, than fluconazole (−8.1 kcal/mol), but less beneficial then posaconazole (−13.2 kcal/mol, Table 2).

**Figure 1:**
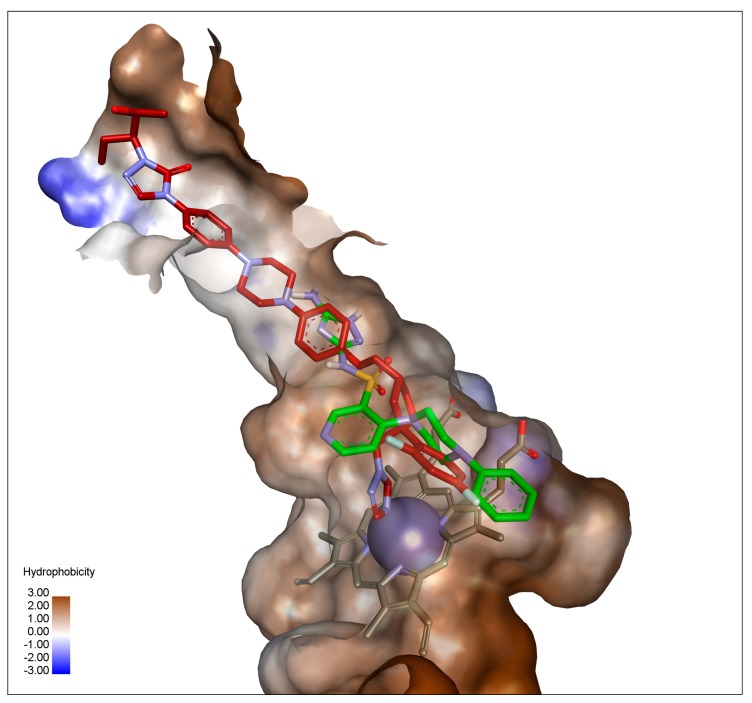
Superimposition of computed binding geometry of compound **26** (green) and conformation of co-crystallized ligand posaconazole (red) inside the solvent accessible surface of CYP51 active site.

**Figure 2:**
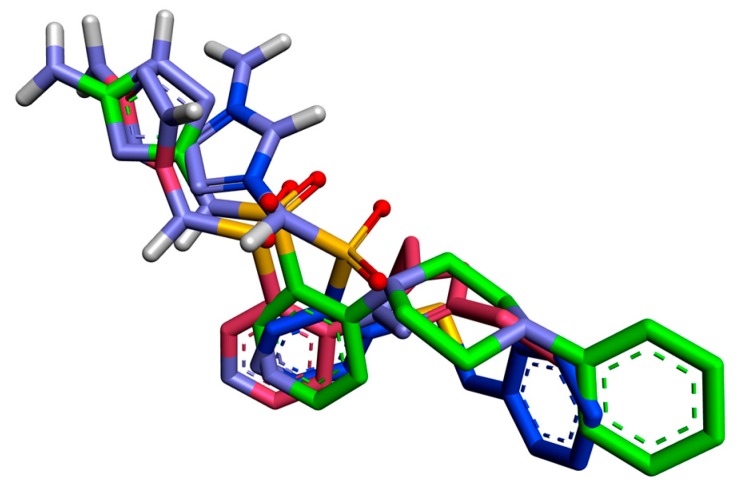
Superimposition of the computed binding geometry of compounds **26** (green), **34** (purple) and **35** (blue). Atoms other than carbon: sulfur—yellow, nitrogen—violet, oxygen—red, hydrogen—grey.

**Figure 3:**
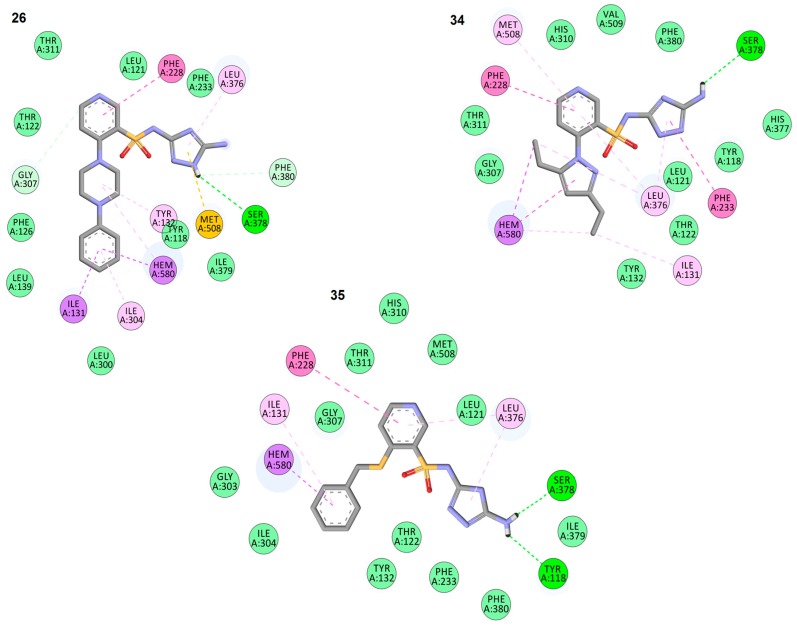
The two-dimensional presentation of enzyme-ligand interaction of compounds **26**, **34** and **35** and the active site of CYP51 (PDB ID:5FSA).

The computed geometries of compounds **26**, **34** and **35** suggest however different binding patterns of the tested compound than that of well-known azole antifungal drugs like fluconazole, and posaconazole [35]. Generally, the 1,2,4-triazole moiety in the investigated compounds doesn’t interact covalently with the CYP51 heme, instead is directed towards hydrophobic tunnel connecting the chamber adjacent to the heme with the protein surface (Figure 1 and Figure 2). Furthermore, the NH or NH_2_ group of the aminotriazole ring of compounds **26**, **34** and **35** forms hydrogen bonds with Ser378. The space over the Fe ion, which supposed to be occupied by the triazole, is filled with the pyridine ring stabilized by a π-π interaction with Phe228 (Table 2, Figure 3).On the other hand the substituent from position 4 of the pyridine scaffold is directed deeply inside the active pocket (previously occupied by the difluorophenyl ring of posaconazole) interacting with the porphyrin ring and active site residues (Figure 1 and Figure 2). Location of substituents from the position 4 in the binding cleft may be responsible for large differences in activity of synthesized compounds correlated with relatively minor changes in the structure of this substituent (e.g., compounds **26** and **29** or **33** and **34**). It is possible to suppose that this low molecular compounds (MW < 470) with simple and modular structure, and easily susceptible to structural diversification with substituents in position 4 of pyridine ring makes the *N*-triazolopyridine sulfonamide scaffold a suitable molecule for further hit to lead optimization in designing anti-*Candida* drugs.

### 2.4. Anticancer Activity

Recently, we have shown that 4-(4-arylpiperazin-1-yl)pyridine-3-sulfonamides **2**–**6** exhibited moderate antitumor activity [15], while some of their sulfonylurea derivatives [13] showed antitumor activity either beneficial or comparable to the clinically tested diarylsulfonylurea sulofenur [16]. This prompted us to evaluate obtained new compounds **20**, **26**, **28**–**31** and **34**–**36** in the NCI-60 Human Tumor Cell Lines Screen in the National Cancer Institute (Bethesda, MD, USA). The preliminary assay was performed at a single high concentration of 10 µM against the NCI panel of 60 cell lines derived from nine different human cancer types: leukemia, non-small-cell lung cancer (NSCLC), colon, central nervous system (CNS), melanoma, ovarian, renal, prostate and breast cancer. In results inhibition growth percent (IGP) compared to no-drug control, have been calculated, (data obtained for the most sensitive cell lines are shown Table S1 in Supplementary Materials). In this series, moderate cytostatic activity (IGP ≥ 10%) of particular compounds, was observed only for 1 to 5 cell lines from whole NCI-60 panel. The highest IGP value of 23% was found for compound **34** (R = 3,5-diethyl-1*H*-pyrazole) towards the MCF7 breast cancer cell line and for compound **36** (IGP = 21%; R = -SCH_2_CONH_2_) towards the SNB-75 brain cancer line. No significant relationship was found between cytostatic activity and the structure of tested compounds, with the exception of increased sensitivity of renal tumor UO-31 (IGP = 10–17%) to compounds **26**, **28**–**30** with 4-phenylpiperazine substituent at the 4-position of the pyridine ring.

## 3. Materials and Methods

### 3.1. General Information

The following instruments and parameters were used: melting points: Boethius HMK apparatus (Veb Analytic, Dresden, Germany); IR spectra: KBr pellets, 400–4000 cm^−1^ Thermo Mattson Satellite FTIR spectrophotometer (Thermo Mattson, Madison, WI, USA); ^1^H- and ^13^C-NMR spectra: Gemini 200 at 200 and 50 MHz (^13^C), respectively, or Unity 500 Plus apparatus at 500 MHz and 125 MHz (^13^C) (Varian, Palo Alto, CA, USA); chemical shifts are expressed at δ in ppm values relative to TMS as standard. LC-MS analyses of compound **37** was performed on a: LCMS-ESI-IT-TOF LC-20A mass spectrometer (Shimadzu Scientific Instruments, Columbia, MD, USA) in positive ion mode. Elemental analyses (C,H,N) were performed on 2400 Series II CHN Elemental Analyzer (Perkin Elmer, Shelton, CT, USA). Thin-layer chromatography was performed on silica gel 60 F254 TLC plates (Merck, Darmstadt, Germany) using benzene/ethanol (4:1) as mobile phases, and visualized with UV light 254 or 366 nm.

Marvin ver. 17.9, was used for drawing, displaying and characterizing chemical structures, substructures and reactions (ChemAxon, http://www.chemaxon.com, 2017).

The starting 4-chloro-3-pyridinesulfonamide (**1**) was commercially available (Alfa Aesar, Karlsruhe, Germany), and primary 4-substituted pyridine-3-sulfonamides **2**–**14** were obtained according to previously described methods (**2**–**11 [15]** and **12**–**14** [13]).

### 3.2. Synthesis

#### 3.2.1. Procedure for the Preparation of Methyl *N*′-Cyano-*N*-{[4-(piperazin-1-yl)pyridin-3-yl]sulfonyl}carbamimidothioates **15**–**19**

A mixture of appropriate 4-(piperazin-1-yl)pyridine-3-sulfonamide (**2**–**6**) (2.5 mmol), dimethyl *N*-cyanodithioiminocarbonate (0.40 g, 2.75 mmol) and anhydrous potassium carbonate (0.33 g, 2.5 mmol) in dry acetone (7.5 mL) was stirred under reflux for 18 h. The reaction mixture was evaporated under reduced pressure, residue was dissolved in water 5 mL, and extracted with dichloromethane (3 × 5 mL). Organic layer was removed and water phase was acidified with diluted hydrochloric acid to pH = 7. The precipitate obtained was collected by filtration and dried. In this manner, the following products were obtained.

*Methyl N′-cyano-N-{[4-(4-phenylpiperazin-1-yl)pyridin-3-yl]sulfonyl}carbamimidothioate* (**15**). Starting from 4-(4-phenylpiperazin-1-yl)pyridine-3-sulfonamide **2** (0.76 g) the title compound was obtained. Yield 0.87 g, (84%); m.p. 241–244 °C; IR (KBr): 3177 (NH), 3092 (C_Ar_-H), 2922 (C-H), 2161 (C≡N), 1645, 1599, 1528 (C=C, C=N) cm^−1^; ^1^H-NMR (500 MHz, DMSO-*d*_6_) δ: 2.36 (s, 3H, S-CH_3_), 3.33 (m, 4H, 2 × CH_2_), 4.07 (m, 4H, 2 × CH_2_), 6.80 (t. 1H, H-4 Ph), 6.98 (d. 2H, H-2,6 Ph), 7.23 (t. 2H, H-3,5 Ph), 7.36 (d. 1H, H-5 pyrid.), 8.31(d. 1H, H-6 pyrid.), 8.82 (s. 1H, H-2 pyrid.), 13.65 (brs, 1H, NH) ppm. Anal. calcd. for C_18_H_20_N_6_O_2_S_2_ (416.52); C, 51.90; H, 4.84; N, 20.18. Found: C, 51.42; H, 4.70; N, 20.17.

*Methyl N-[4-[4-(4-chlorophenyl)piperazin-1-yl]pyridin-3-yl}sulfonyl-N′-cyanocarbamimidothioate* (**16**). Starting from 4-[4-(4-chlorophenyl)piperazin-1-yl]pyridine-3-sulfonamide **3** (0.88 g) the title compound was obtained. Yield 1.01 g, (90%); m.p. 254–257 °C dec.; IR (KBr): 3172 (NH), 3090 (C_Ar_-H), 2987, 2924 (C-H), 2162 (C≡N), 1647, 1572, 1486 (C=C, C=N), 1326, 1127 (SO_2_) cm^−1^; ^1^H-NMR (500 MHz, DMSO-*d*_6_) δ: 2.36 (s, 3H, S-CH_3_), 3.34 (m, 4H, 2 × CH_2_), 4.06 (m, 4H, 2 × CH_2_), 7.00 (d, 2H, H-2, 6 Ph), 7.25 (d, 2H, H-3,5 Ph), 7.36 (d, 1H, H-5 pyrid.), 8.30 (d, 1H, H-6 pyrid.), 8.81 (s, 1H, H-2 pyrid.), 13.74 (brs, 1H, NH) ppm; ^13^C-NMR (DMSO-*d*_6_, 50 MHz) δ: 15.60; 47.69; 50.55; 113.53; 116.50; 116.99; 122.89; 126.19; 128.97; 139.97; 145.53; 149.30; 156.03; 173.86 ppm. Anal. calcd. for C_18_H_19_ClN_6_O_2_S_2_ (450.97); C, 47.94; H, 4.25; N, 18.64. Found: C, 47.76; H, 4.14; N, 18.30.

*Methyl N′-cyano-N-{[4-[4-(4-fluorophenyl)piperazin-1-yl]pyridin-3-yl]sulfonyl}carbamimidothioate* (**17**). Starting from 4-[4-(4-fluorophenyl)piperazin-1-yl]pyridine-3-sulfonamide **4** (0.84 g) the title compound was obtained. Yield 0.76 g, (70%); m.p. 248–251 °C; IR (KBr): 3175 (NH), 3088 (C_Ar_-H), 2925, 2850 (C-H), 2161 (C≡N), 1646, 1570, 1509 (C=C, C=N), 1301, 1128 (SO_2_) cm^−1^; ^1^H-NMR (500 MHz, DMSO-*d*_6_) δ: 2.35 (s, 3H, S-CH_3_), 3.26 (brs, 4H, 2 × CH_2_), 4.04 (brs, 4H, 2×CH_2_), 6.99 (m, 2H, H-2,6 Ph), 7.06 (t, 2H, H-3,5 Ph), 7.35 (d, 1H, H-5 pyrid.), 8.29 (d. 1H, H-6 pyrid.), 8.80 (s, 1H, H-2 pyrid.), 13.75 (brs, 1H, NH) ppm. Anal. calcd. for C_18_H_19_FN_6_O_2_S_2_ (434,51); C, 49.76; H, 4.41; N, 19.34. Found: C, 49.72 H, 4.36; N, 19.40.

*Methyl N′-cyano-N-{[4-[4-(3,4-dichlorophenyl)piperazin-1-yl]pyridin-3-yl]sulfonyl}carbamimidothioate* (**18**). Starting from 4-[4-(3,4-dichlorophenyl)piperazin-1-yl]pyridine-3-sulfonamide **5** (0.97 g) the title compound was obtained. Yield 0.82 g, (68%); m.p. 251–254 °C; IR (KBr): 3167 (NH), 3093 (C_Ar_-H), 2925, 2843 (C-H), 2161 (C≡N), 1648, 1594, 1570, 1480 (C=C, C=N), 1329, 1125 (SO_2_) cm^−1^; ^1^H-NMR (500 MHz, DMSO-*d*_6_) δ: 2.35 (s, 3H, S-CH_3_), 3.40 (brs, 4H, 2 × CH_2_), 4.04 (brs, 4H, 2 × CH_2_), 6.95 (dd, 1H, H-6 Ph) 7.17 (d, 1H, H-2 Ph), 7.33 (d, 1H, H-5 pyrid.), 7.40 (d, 1H, H-5 Ph), 8.29 (d. 1H, H-6 pyrid.), 8.81 (s, 1H, H-2 pyrid.), 13.80 (brs, 1H, NH) ppm. Anal. calcd. for C_18_H_18_Cl_2_N_6_O_2_S_2_ (485.41); C, 44.54; H, 3.74; N, 17.31. Found: C, 44.69; H, 3.50; N, 17.31.

*Methyl N′-cyano-N-{[4-[4-(2-methoxyphenyl)piperazin-1-yl]pyridin-3-yl]sulfonyl}carbamimidothioate* (**19**). Starting from 4-[4-(2-methoxyphenyl)piperazin-1-yl]pyridine-3-sulfonamide **6** (0.87 g) the title compound was obtained. Yield 0.81 g, (73%); m.p. 238–240 °C; IR (KBr): 3198 (NH), 3032 (C_Ar_-H), 2964, 2918, 2850 (C-H), 2168 (C≡N), 1639, 1596, 1501, 1483 (C=C, C=N), 1341, 1146 (SO_2_) cm^−1^; ^1^H-NMR (500 MHz, DMSO-*d*_6_) δ: 2.35 (s, 3H, S-CH_3_), 3.13 (s, 4H, 2 × CH_2_), 3.80 (s, 3H, CH_3_), 4.03 (s, 4H, 2 × CH_2_), 6.89–6.97 (m, 4H, Ph.), 7.35 (d, 1H, H-5 pyrid.), 8.29 (d. 1H, H-6 pyrid.), 8.80 (s, 1H, H-2 pyrid.), 13.60 (brs, 1H, NH) ppm. Anal. calcd. for C_19_H_22_N_6_O_3_S_2_ (446.55); C, 51.10; H, 4.97; N, 18.82. Found: C, 50.73; H, 4.88 N, 18.41.

#### 3.2.2. Procedure for the Preparation of Methyl *N*-(Pyridin-3-yl)sulfonyl-*N*′-cyanocarbamimidothioate **20**–**25**

A mixture of the appropriate 4-substituted pyridine-3-sulfonamide **8**–**11**,**13**,**14** (2.5 mmol), dimethyl *N*-cyanodithioiminocarbonate (0.40 g, 2.75 mmol) and anhydrous potassium carbonate (0.33 g, 2.5 mmol) in dry acetone (7.5 mL) was stirred under reflux for 12 h. The precipitate was collected by filtration, and dried, then suspended in water (2.5 mL) and slowly adjusted to pH 2 with 4% solution of HCl (analogously—adjusting suspension to pH 7 with 4% solution of HCl results in potassium salts **20A**–**25A**). After stirring at room temperature for 2 h, the precipitate was filtered off, washed with cold water (2 × 1 mL), and dried. In this manner, the following products were obtained.

*Methyl N′-cyano-N-{[4-(3,5-dimethyl-1H-pyrazol-1-yl)pyridin-3-yl]sulfonyl}carbamimidothioate* (**20**). Starting from 4-(3,5-dimethyl-1*H*-pyrazol-1-yl)pyridine-3-sulfonamide **8** (0.63 g) the title compound was obtained. Yield 0.63 g, (72%); m.p. 172–175 °C; IR (KBr): 3501 (NH), 3090 (C_Ar_-H), 2930 (C-H), 2162 (C≡N), 1628, 1477 (C=C, C=N), 1289, 1147 (SO_2_) cm^−1^; ^1^H-NMR (500 MHz, DMSO-*d*_6_) δ: 2.04 (s, 3H, CH_3_), 2.13 (s, 3H, CH_3_), 2.16 (s, 3H, CH_3_), 6.06 (s, 1H, H-4 pyrazole), 7.47 (d, 1H, H-5 pyrid.), 8.84 (d, 1H, H-6 pyrid.), 9.12 (s, 1H, H-2 pyrid.) ppm. Anal. calcd. for C_13_H_14_N_6_O_2_S_2_ (350.42); C, 44.56; H, 4.03; N, 23.98. Found: C, 44.26; H, 3.87; N. 23.65.

*Methyl N′-cyano-N-{[4-(3,4,5-trimethyl-1H-pyrazol-1-yl)pyridin-3-yl]sulfonyl}carbamimidothioate* (**21**). Starting from 4-(3,4,5-trimethyl-1*H*-pyrazol-1-yl)pyridine-3-sulfonamide **9** (0.66 g) the title compound was obtained. Yield 0.57 g, (63%); m.p. 181–183 °C; IR (KBr): 3087 (C_Ar_-H), 2925, 2854 (C-H), 2167 (C≡N), 1573, 1470 (C=N), 1302, 1152, 1147 (SO_2_) cm^−1^; ^1^H-NMR (500 MHz, DMSO-*d*_6_) δ: 1.93 (s, 3H, CH_3_), 1.99 (s, 3H, CH_3_), 2.15 (s, 3H, CH_3_), 2.16 (s, 3H, CH_3_), 7.55 (d, 1H, H-5 pyrid.), 8.88 (d, 1H, H-6 pyrid.), 9.14 (s. 1H, H-2 pyrid.) ppm. Anal. calcd. for C_14_H_16_N_6_O_2_S_2_ (364.45); C, 46.14; H, 4.43; N, 23.06. Found: C, 46.30; H, 4.15; N, 22.91.

*Methyl N-[4-(4-butyl-3,5-dimethyl-1H-pyrazol-1-yl)pyridin-3-yl]sulfonyl-N′-cyanocarbamimidothioate* (**22**). Starting from 4-(4-butyl-3,5-dimethyl-1*H*-pyrazol-1-yl)pyridine-3-sulfonamide **10** (0.77 g) the title compound was obtained. Yield 0.66 g, (65%); m.p. 119–123 °C; IR (KBr): 3503 (NH), 3090 (C_Ar_-H), 2961, 2923, 2853 (C-H), 2161 (C≡N), 1621, 1501,1480 (C=C, C=N), 1328, 1148 (SO_2_) cm^−1^; ^1^H-NMR (200 MHz, DMSO-*d*_6_) δ: 0.91 (t, 3H, CH_3_), 1.36 (m, 4H, 2 × CH_2_), 1.95 (s, 3H, CH_3_), 2.10 (s, 3H, CH_3_), 2.15 (s, 3H, CH_3_), 2.34 (t, 2H, CH_2_), 7.29 (d, 1H, H-5 pyrid.), 8.77 (d, 1H, H-6 pyrid.), 9.09 (s, 1H, H-2 pyrid.) ppm; ^13^C-NMR (DMSO-*d*_6_, 50 MHz) δ: 10.08; 12.08; 14.18; 15.30; 22.06; 22.95; 32.63; 116.54; 117.08; 124.71; 137.15; 137.86; 144.45; 146.76; 151.27; 152.85; 173.35 ppm. Anal. calcd. for C_17_H_22_N_6_O_2_S_2_ (406.53); C, 50.23; H, 5.45; N, 20.67. Found: C, 50.02; H, 5.21; N, 20.82.

*Methyl N′-cyano-N-{[4-(3,5-diethyl-1H-pyrazol-1-yl)pyridin-3-yl]sulfonyl}carbamimidothioate* (**23**). Starting from 4-(3,5-diethyl-1*H*-pyrazol-1-yl)pyridine-3-sulfonamide **11** (0.70 g) the title compound was obtained. Yield 0.68 g (72%); m.p. 186–189 °C (MeOH); IR (KBr): 3442, 3137 (NH), 3057 (C_Ar_-H), 2926 (C-H), 2210, 2178 (C≡N), 1571,1474 (C=C, C=N), 1239, 1150 (SO_2_) cm^−1^; ^1^H-NMR (200 MHz, DMSO-*d*_6_) δ: 1.09 (t, 3H, CH_3_), 1.20 (t, 3H, CH_3_), 2.17 (s, 3H, S-CH_3_), 2.38 (q, 2H, CH_2_), 3.56 (q, 2H, CH_2_), 6.15 (s, 1H, H-4 pyrazole), 7.49 (d, 1H, H-5 pyrid), 8.84 (d, 1H, H-6 pyrid.), 9.14 (s, 1H, H-2 pyrid.) ppm. Anal. calcd. for C_15_H_18_N_6_O_2_S_2_ (378.47); C, 47.60; H, 4.79; N, 22.21. Found: C, 47.12; H, 4.58 N, 22.03.

*Methyl N-[4-(benzylthio)pyridin-3-yl]sulfonyl-N′-cyanocarbamimidothioate* (**24**). Starting from 4-(benzylthio)pyridine-3-sulfonamide **13** (0.70 g) the title compound was obtained. Yield 0.57 g; (60%); m.p. 193–194 °C; IR (KBr): 3118 (NH), 2926 (C-H), 2172 (C≡N), 1625, 1494 (C=N, C=C), 1332, 1144 (SO_2_) cm^−1^; ^1^H-NMR (200 MHz, DMSO-*d*_6_) δ: 2.34 (s, 3H, CH_3_), 4.57 (s, 2H, CH_2_), 7.30–7.49 (m, 5H, benzene), 7.88 (d, 1H, H-5 pyrid), 8.61 (d, 1H, H-6 pyrid.), 8.87 (s, 1H, H-2 pyrid.) ppm. Anal. calcd. for C_15_H_14_N_4_O_2_S_3_ (378.49); C, 47.60; H, 3.73; N, 14.80. Found: C, 47.67; H, 3.64; N, 14.52.

*Methyl N-{4-[(2-amino-2-oxoethyl)thio]pyridin-3-yl}sulfonyl-N′-cyanocarbamimidothioate* (**25**). Starting from 2-[(3-sulfamoylpyridin-4-yl)thio]acetamide **14** (0.62 g) the title compound was obtained. Yield 0.63 g, (73%); m.p. 217–219 °C; IR (KBr): 3374, 3269, 3177 (NH), 2922 (C-H), 2170 (C≡N), 1697 (C=O), 1619, 1570, 1480 (C=C, C=N), 1333, 1146 (SO_2_) cm^−1^; ^1^H-NMR (500 MHz, DMSO-*d*_6_) δ: 2.36 (s, 3H, SCH_3_), 3.97 (s, 2H, CH_2_), 7.37 (s, 1H, NH), 7.72 (s, 1H, NH), 7.81 (d, 1H, H-5 pyrid.), 8.62 (d, 1H, H-6 pyrid.), 8.86 (s, 1H, H-2 pyrid.) ppm. Anal. calcd. for C_10_H_11_N_5_O_3_S_3_ (345.42); C, 34.77; H, 3.21; N, 20.27. Found: C, 34.38; H, 3.09; N, 20.33.

#### 3.2.3. Procedure for the Preparation of *N*-(5-Amino-1*H*-1,2,4-triazol-3-yl)-4-(piperazin-1-yl)pyridine-3-sulfonamides **26**–**30**

Potassium methyl *N*′-cyano-*N*-{[4-(piperazin-1-yl)pyridin-3-yl]sulfonyl} carbamimidothioates **15**–**19** (1 mmol) were dissolved in 1% KOH (5.5 mL), stirred for 15 min and then then water was evaporated under reduced pressure. The residue was dissolved in anhydrous acetonitrile (4 mL) and hydrazine monohydrate (0.25 g, 5 mmol) was added. Next the mixture was refluxed for 7–16 h. The precipitate obtained was collected by filtration, suspended in water (4 mL) and acidified to pH 6 with 4% hydrochloric acid. After stirring for a few hours the solid was filtered off, washed with cold water and dried. In this manner, the following products were obtained:

*N-(5-Amino-1H-1,2,4-triazol-3-yl)-4-(4-phenylpiperazin-1-yl)pyridine-3-sulfonamide* (**26**). Starting from methyl *N*′-cyano-*N*-{[4-(4-phenylpiperazin-1-yl)pyridin-3-yl]sulfonyl}carbamimidothioate (**15**, 0.42 g) and refluxing for 3.5 h the title compound was obtained. Yield 0.26 g, (66%); m.p. 185–187 °C; IR (KBr): 3438, 3344 (NH), 2924, 2847 (C-H), 1588 (C=C, C=N), 1328, 1141 (SO_2_) cm^−1^; ^1^H-NMR (200 MHz, DMSO-*d*_6_) δ: 3.24 (s, 4H, 2 × CH_2_), 3.45 (s, 4H, 2 × CH_2_), 5.86 (brs, 2H, NH_2_), 6.79 (t, 1H, H-4 Ph), 6.97 (d, 2H, H-2,6 Ph), 7.10 (d, 1H, H-4 pyrid.), 7.23 (t, 2H, H-3,5 Ph), 8.41(d, 1H, H-6 pyrid.), 8.91(s, 1H, H-2 pyrid.), 11.50 (brs, 1H, NH), 11.95 (brs, 1H, NH) ppm. Anal. calcd. for C_17_H_20_N_8_O_2_S (400.46); C, 50.99; H, 5.03; N, 27.98. Found: C, 50.74; H, 5.04; N, 27.61.

*N-(5-Amino-1H-1,2,4-triazol-3-yl)-4-[4-(4-chlorophenyl)piperazin-1-yl]pyridine-3-sulfonamide* (**27**). Starting from methyl *N*-[4-[4-(4-chlorophenyl)piperazin-1-yl]pyridin-3-yl}sulfonyl-*N*′-cyano-carbamimidothioate (**16**) (0.45 g) and refluxing for 16 h the title compound was obtained. Yield 0.22 g, (50%); m.p. 162 °C dec; IR (KBr): 3433, 3334 (NH), 2911, 2844 (C-H), 1654, 1636, 1597 (C=C, C=N), 1387,1149 (SO_2_) cm^−1^; ^1^H-NMR (200 MHz, DMSO-*d*_6_) δ: 3.25 (s, 4H, 2 × CH_2_), 3.46 (s, 4H, 2 × CH_2_), 5.87 (brs, 2H, NH_2_), 6.99 (d, 2H, H-2,6 Ph), 7.12 (d, 1H, H-5 pyrid.), 7.26 (d, 2H, H-3,5 Ph), 8.43 (d, 1H, H-6 pyrid.), 8.92 (s, 1H, H-2 pyrid.), 11.50 (brs, 1H, NH), 12.00 (brs, 1H, NH) ppm;^13^C-NMR (DMSO-*d*_6_, 125 MHz) δ: 48.27; 51.07; 115.14; 117.42; 122.84; 129.04; 131.23; 148.89; 150.12; 150.52; 150.53; 152.49; 155.81 ppm. Anal. calcd. for C_17_H_19_ClN_8_O_2_S (434.90); C, 46.95; H, 4.40; N, 25.77. Found: C, 46.91; H, 4.38; N, 25.70.

*N-(5-Amino-1H-1,2,4-triazol-3-yl)-4-[4-(4-fluorophenyl)piperazin-1-yl]pyridine-3-sulfonamide* (**28**). Starting from methyl *N*′-cyano-*N*-{[4-[4-(4-fluorophenyl)piperazin-1-yl]pyridin-3-yl]sulfonyl}-carbamimidothioate (**17**, 0.43 g) and refluxing for 3.5 h the title compound was obtained. Yield 0.22 g, (54%); m.p. 165–168 °C; IR (KBr): 3446, 3361, 3190 (NH), 2841 (C-H), 1611,1561 (C=C, C=N), 1384,1149 (SO_2_) cm^−1^; ^1^H-NMR (500 MHz, DMSO-*d*_6_) δ: 3.16 (s, 4H, 2×CH_2_), 3.43 (s, 4H, 2×CH_2_), 5.82 (brs, 2H, NH_2_), 6.96–6.99 (m, 2H, H-2,6 Ph), 7.03–7.09 (m, 4H, H-3,5 Ph, H-5 pyrid.), 8.41 (d, 1H, H-6 pyrid.), 8.9 (s. 1H, H-2 pyrid.), 11.41 (brs, 1H, NH), 11.90 (brs, 1H, NH) ppm. Anal. calcd. for C_17_H_19_FN_8_O_2_S (418.45); C, 48.79; H, 4.58; N, 26.78. Found: C, 48.66, H, 4.88; N, 26.57.

*N-(5-Amino-1H-1,2,4-triazol-3-yl)-4-[4-(3,4-dichlorophenyl)piperazin-1-yl]pyridine-3-sulfonamide* (**29**). Starting from methyl *N*′-cyano-*N*-{[4-[4-(3,4-dichlorophenyl)piperazin-1-yl]pyridin-3-yl]sulfonyl}-carbamimidothioate (**18**, 0.48 g) and refluxing for 3.5 h the title compound was obtained. Yield 0.19 g, (40%); m.p. 175–178 °C dec; IR (KBr): 3495, 3380 (NH), 2922, 2851 (C-H), 1696, 1643, 1591 (C=C, C=N), 1384,1141 (SO_2_) cm^−1^; ^1^H-NMR (200 MHz, DMSO-*d*_6_) δ: 3.36 (s, 4H, 2 × CH_2_), 4.04 (s, 4H, 2 × CH_2_), 6.05 (brs, 2H, NH_2_), 6.98 (m, 2H, H-2,6 Ph), 7.28 (m, 2H, H-5 pyrid. H-5 Ph), 8.34 (d, 1H, H-6 pyrid.), 8.89 (s, 1H, H-2 pyrid. 12.10 (brs, 2H, 2 × NH) ppm. Anal. calcd. for C_17_H_18_Cl_2_N_8_O_2_S (469.35); C, 43.50; H, 3.87; N, 23.87. Found: C, 43.22; H, 3.81; N, 23.62.

*N-(5-Amino-1H-1,2,4-triazol-3-yl)-4-[4-(2-methoxyphenyl)piperazin-1-yl]pyridine-3-sulfonamide* (**30**). Starting from methyl *N*′-cyano-*N*-{[4-[4-(2-methoxyphenyl)piperazin-1-yl]pyridin-3-yl]sulfonyl}-carbamimidothioate (**19**, 0.45 g) and refluxing for 3.5 h the title compound was obtained. Yield 0.23 g, (54%); m.p. 176–178 °C dec; IR (KBr) : 3420, 3329 (NH), 2941, 2884, 2839 (C-H), 1621, 1585 (C=C, C=N), 1375, 1141 (SO_2_), 1241 (C-O) cm^−1^; ^1^H-NMR (200 MHz, DMSO-*d*_6_) δ: 3.04 (s, 4H, 2 × CH_2_), 3.44 (s, 4H, 2 × CH_2_), 3.79 (s, 3H, CH_3_), 5.87 (brs, 2H, NH_2_), 6.93 (m, 4H, Ph), 7.12 (d, 1H, H-5 pyrid.), 8.41 (d, 1H, H-6 pyrid.), 8.91 (s, 1H, H-2 pyrid.), 11.45 (brs, 1H, NH), 11.90 (brs, 1H, NH) ppm. Anal. calcd. for C_18_H_22_N_8_O_3_S (430.48); C, 50.22; H, 5.15; N, 26.03. Found: C, 50.03; H, 4.98; N, 25.60.

#### 3.2.4. Procedure for the Preparation of *N*-(5-Amino-1*H*-1,2,4-triazol-3-yl)-4-*R*-pyridine-3-sulfonamide **31**–**36**

To the corresponding potassium [(cyanoimino)(methylthio)methyl][(4-substitutedpyridin-3-yl)sulfonyl]amide **20A**–**25A** (1 mmol) in anhydrous ethanol (2.5 mL, hydrazine monohydrate (0.25 g, 5 mmol) was added and mixture was refluxed for 3.5 h (compounds **31**–**34** and **36**) or stirred at room temperature for 24 h (compound **35**). The precipitate was collected by filtration, suspended in water (3 mL) and acidified o pH 6 with 4% hydrochloric acid. After stirring for a few hours the solid was filtered off, washed with cold water and dried. In this manner, the following products were obtained:

*N-(5-Amino-1H-1,2,4-triazol-3-yl)-4-(3,5-dimethyl-1H-pyrazol-1-yl)pyridine-3-sulfonamide* (**31**). Starting from potassium salt **20A** (0.39 g) 0.20 g (60%) of the title compound were obtained; m.p. 230 °C dec; IR (KBr): 3524, 3404 (NH), 2925 (C-H), 1678, 1594 (C=C, C=N), 1276, 1158 (SO_2_) cm^−1^; ^1^H-NMR (200 MHz, DMSO-*d*_6_) δ: 2.04 (s, 6H, 2 × CH_3_), 5.84 (s. 2H, NH_2_), 5.93 (s, 1H, H-4, pyrazole), 7.36 (d, 1H, H-5, pyrid.), 8.83 (d, 1H, H-6 pyrid.), 9.19 (s, 1H, H-2 pyrid.), 11.00 (brs, 1H, NH), 11.90 (brs, 1H, NH) ppm; ^13^C-NMR (DMSO-*d*_6_, 125 MHz) δ: 11.89; 13.78; 103.86; 106.75; 125.51; 141.78; 144.30; 149.22; 150.51; 154.14 ppm. Anal. calcd. for C_12_H_14_N_8_O_2_S (334.36); C, 43.11; H, 4.22; N, 33.51. Found: C, 42.82; H, 4.46; N, 33.16.

*N-(5-Amino-1H-1,2,4-triazol-3-yl)-4-(3,4,5-trimethyl-1H-pyrazol-1-yl)pyridine-3-sulfonamide* (**32**). Starting from potassium salt **21A** (0.44 g) 0.17 g (48%) of the title compound were obtained; m.p. 217–219 °C; IR (KBr): 3417 (NH), 2923, 2854 (C-H), 1628, 1584 (C=C, C=N), 1293, 1153 (SO_2_) cm^−1^; ^1^H-NMR (200 MHz, DMSO-*d_6_*) δ: 1.86 (s, 3H, CH_3_), 1.96 (s, 3H, CH_3_), 1.99 (s. 3H, CH_3_), 5.82 (s. 2H, NH_2_), 7.29 (d. 1H, H-5 pyrid.), 8.81 (d. 1H, H-6 pyrid.), 9.18 (s. 1H, H-2 pyrid.), 10.84 (br. s. 1H, NH), 11.87 (br. s. 1H, NH) ppm. Anal. calcd. for C_13_H_16_N_8_O_2_S (348.38); C, 44.82; H, 4.63; N, 32.16. Found: C, 44.61; H, 4.61; N, 31.81.

*N-(5-Amino-1H-1,2,4-triazol-3-yl)-4-(4-butyl-3,5-dimethyl-1H-pyrazol-1-yl)pyridine-3-sulfonamide* (**33**). Starting from potassium salt **22A** (0.44 g) 0.114 g, (42%) of the title compound were obtained; m.p. 215 °C dec; IR (KBr): 3422, 3325, 3277, 3215(NH), 2958, 2928, 2860 (C-H), 1660, 1630, 1584 (C=C, C=N), 1298, 1155 (SO_2_) cm^−1^; ^1^H-NMR (200 MHz, DMSO-*d*_6_) δ: 0.92 (t, 3H, CH_3_), 1.34 (m, 4H, 2×CH_2_), 1.99 (s, 3H, CH_3_), 2.03 (s, 3H, CH_3_), 2.31 (t, 2H, CH_2_), 5.83 (s, 2H, NH_2_), 7.34 (d, 1H, H-5 pyrid.), 8.82 (d, 1H, H-6 pyrid.), 9.19 (s, 1H, H-2 pyrid.), 11.00 (s, 1H, NH), 11.91 (s, 1H, S-NH)Anal. calcd. for C_16_H_22_N_8_O_2_S (390.46); C, 49.22; H, 5.68; N, 28.70. Found C, 49.47; H, 5.49; N, 28.77.

*N-(5-Amino-1H-1,2,4-triazol-3-yl)-4-(3,5-diethyl-1H-pyrazol-1-yl)pyridine-3-sulfonamide* (**34**). Starting from potassium salt **23A** (0.42 g) 0.16 g, (45%) of the title compound were obtained; m.p. 223 °C dec; IR (KBr): 3445, 3371 (NH), 2973, 2924 (C-H), 1644, 1587 (C=C, C=N), 1279, 1147 (SO_2_) cm^−1^; ^1^H-NMR (200 MHz, DMSO-*d*_6_) δ: 1.06 ( t, 3H, CH_3_), 1.12 (t, 3H, CH_3_), 2.37 (q, 2H, CH_2_), 2.45 (q, 2H, CH_2_), 5.82 (s, 2H, NH_2_), 6.00 (s, 1H, H-4 pirazol), 7.37 (d, 1H, H-5 pyrid.), 8.81 (d, 1H, H-6 pyrid.), 9.17 (s, 1H, H-2 pyrid.), 11.07 (s, 1H, NH), 11.92 (s, 1H, S-NH) ppm. Anal. calcd. for C_14_H_18_N_8_O_2_S (362.41); C, 46.40; H, 5.01; N, 30.92. Found: C, 45.91; H, 4.88 N, 30.98.

*N-(5-Amino-1H-1,2,4-triazol-3-yl)-4-(benzylthio)pyridine-3-sulfonamide* (**35**). Starting from potassium salt (**24A**) (0.42 g) the title compound was obtained 0.21 g, (59%); m.p. 240 °C dec; IR (KBr): 3454, 3357 (NH), 3065 (C_ar_-H), 2917 (C-H), 1620, 1574 (C=C, C=N), 1144 (SO_2_) cm^−1^; ^1^H NMR (200 MHz, DMSO-*d*_6_) δ: 4.35 (s, 2H, S-CH_2_), 5.87 ( brs, 2H, NH_2_), 7.27–7.49 (m, 6H, Ph, H-5 pyrid.), 8.45 (d, 1H, H-6 pyrid.), 8.87 (s, 1H, H-2 pyrid.), 11.60 (brs, 1H, NH), 11.99 (brs, 1H, NH) ppm. Anal. calcd. for C_14_H_14_N_6_O_2_S_2_ (362.43); C, 46.60; H, 3.89; N, 23.19. Found: C, 46.41; H, 3.78; N, 23.43.

*2-{[3-[N-(5-Amino-1H-1,2,4-triazol-3-yl)sulfamoyl]pyridin-4-yl]thio}acetamide* (**36**). Starting from potassium salt **25A** (0.38 g) 0.23 g, (69%) of the title compound were obtained; m.p. 220 °C dec; IR (KBr): 3338, 3191 (NH), 2924 (C-H), 1682 (C=O), 1586 (C=N, C=C), 1329, 1149 (SO_2_) cm^−1^; ^1^H-NMR (500 MHz, DMSO-*d*_6_) δ: 3.74 (s, 2H, CH_2_), 5.86 (brs, 2H, NH_2_), 7.28 (brs, 1H, NH amid), 7.42 (d, 1H, H-5 pyrid.), 7.65 (brs, 1H, NH amide), 8.47 (d, 1H, H-6 pyrid.), 8.87 (s, 1H, H-2 pyrid.), 11.63 (brs, 1H, NH), 12.03 (brs,1H, NH) ppm. Anal. calcd. for C_9_H_11_N_7_O_3_S_2_ (329.36), C, 32.82; H, 3.37; N, 29.77. Found: C, 32.72, H, 3.33. N, 29.74.

#### 3.2.5. Procedure for the Preparation of *N*-(5-Amino-1*H*-1,2,4-triazol-3-yl)-4-hydrazinylpyridine-3-sulfonamide **37**

To methyl *N*-(pyridin-3-yl)sulfonyl-*N*′-cyanocarbamimidothioate **20**–**23** (1 mmol) in anhydrous ethanol (3.5 mL), hydrazine monohydrate (0.25 g, 5 mmol) was added and the mixture was refluxed for 5.5 h. The precipitate was collected by filtration, and purified by crystallization from 75% MeOH. Yield 0.10–0.17 g, (32–37%); m.p. 207–211 °C; IR (KBr): 3408, 3375, 3317, 3259, 3198(NH), 1659, 1614, 1598 (C=C, C=N), 1285, 1144 (SO_2_) cm^−1^; ^1^H-NMR (200 MHz, DMSO-*d*_6_) δ: 4.60 (brs, 2H, NH_2_), 5.83 (brs, 2H, NH_2_), 5.86 (brs, 1H, NH), 7.10 (d, 1H, H-5 pyrid.), 7.65 (brs, 1H, NH), 8.15(d, 1H, H-6 pyrid.), 8.48(s, 1H, H-2 pyrid.), 11.72 (brs, 1H, NH), ppm. ^13^C-NMR (DMSO-*d_6_*, 50 MHz) δ: 106.28; 120.29; 147.14; 149.41; 151.18; 151.82; 152.57 ppm.LC-MS (ESI) IT-TOF *m*/*z,* calcd for C_7_H_10_N_8_O_2_S: 270.065, found: [M + H]^+^ 271.058.Anal. calcd. for C_7_H_10_N_8_O_2_S (270.27); C, 31.11; H, 3.73; N, 41.46. Found C, 30.83; H, 3.60; N, 41.10.

### 3.3. Antifungal Evaluation

The strains used in assay were isolated from patients with candidiasis of the oral cavity and respiratory tract and were identified by standard morphological and biochemical methods (API tests-system, bioMerieux, Durham, NC, USA) [36,37]. The investigation included 31 clinical strains belonging to the genera of *Candida* (26 strains), *Geotrichum* (2), *Rhodotorula* (2) and *Saccharomyces* (1) and 6 standardized strains: *Candida albicans* ATCC 10231, *Candida glabrata* ATCC 66032, *Candida krusei* ATCC 14243, *Candida lusitaniae* ATCC 34499, *Candida parapsilosis* ATCC 22019 and *Candida tropicalis* ATCC 750 (the reference strains were obtained from the American Type Culture Collection (ATCC, Rockville, MD, USA)). The susceptibility (MIC) of fungi was determined by means of the dilution technique in the Sabouraud’s agar. The compounds were dissolved in 1 mL of dimethylsulfoxide (DMSO) right before the experiments. Further dilutions were performed using sterile distilled water giving final concentrations: 200, 100, 50, 25, 12.5 and 6.2 µg/mL. Fluconazole (Fluka, Buchs, Switzerland) was used as a reference antifungal drug (in concentrations ranging from 3.1 to 100 µg/mL). Appropriate concentrations of each compound or fluconazole were added to Sabouraud’s agar. Inocula containing 10^5^ colony forming units (CFU) per spot was seeded with a Steers replicator applied on the surface of the agar. The agar plates were incubated under aerobic conditions for 24 h at 37 °C. The MIC was defined as the lowest concentration of the compound that completely inhibited the growth of tested fungi. 

### 3.4. Molecular Docking

The chemical structures of the compounds **26**, **34**, **35,** fluconazole and posaconazole were drawn in the Avogadro software and their geometry was optimized by the MMF94 method. Further ligand preparation including addition of Gasteiger charges, merging non-polar hydrogen atoms, and preparing .pdbqt input files was performed using AutoDock Tools 1.5.7 program (ADT) [38]. The crystal structure of the cytochrome P450 sterol 14α-demethylase (CYP51) from *Candida Albicans*(PDB deposited code: 5FSA) was taken from Protein Data Bank [33]. Co-crystallized ligand - posaconazole and water molecules were removed from the initial protein structure and all docking procedures were performed on chain A. Using AutoDock Tools, polar hydrogen atoms were added, non-polar hydrogen atoms merged, and Gasteiger partial charges were assigned. Charge for Fe^2+^ ion in heme structure was added manually in .pdbqt input file. The center of grid box was set *x* = 193.6, *y* = 1.0, *z* = 39.0 and the grid box size was set to *x* = 28 Å, *y* = 32 Å, *z* = 24 Å. The docking calculations were done by Autodock Vina (v. 1.1.2) with the exhaustiveness level of 8 [34]. For each compound, AutoDock Vina searched for 10 conformers, and top ranked conformation with highest binding affinity was analyzed. Interactions were identified and the figures were prepared using the Discovery Studio Visualizer 2016 (Accelerys, San Diego, CA, USA).

### 3.5. Antitumor Evaluation

Antitumor evaluation of compounds **20**, **26**, **28**–**31** and **34**–**36** was performed at the National Cancer Institute (Bethesda, MD, USA). Sulforhodamine B (SRB) assay was performed according to NCI-60 DTP Human Tumor Cell Line Screen procedure [39,40].

## 4. Conclusions

A new series of *N*-(5-amino-1*H*-1,2,4-triazol-3-yl)pyridine-3-sulfonamide derivatives **26**–**36** have been synthesized by the reactions of methyl *N*′-cyano-*N*-[(pyridin-3-yl)sulfonyl]carbamimido-thioates potassium salts **15A**–**25A**, with hydrazine hydrate. It has been found that treatment of the protonated form of methyl *N*′-cyano-*N*-[(pyridin-3-yl)sulfonyl]carbamimidothioates **20**–**23** with hydrazine hydrate results in substitution of 1*H*-pyrazole ring in position 4 with a hydrazine moiety.

Antifungal studies revealed the high sensitivity of *Candida albicans* strains towards the tested compounds, as well as significant efficacy of individual compounds against *Rhodotorula mucilaginosa* (compounds **26**, **28**, **35***)*, *Candida glabrata* (compound **36**), *Candida guilliermondii* (compound **26**), *Candida kruseic* (compound **35**), and *Candida tropicalis* (compound **31***)*. Compounds **26** and **35** were the most active antifungal agents against *Candida albicans* and *Rhodotorula mucilaginosa* species, with their MIC ≤ 6.2 µg/mL being more potent than that of fluconazole.

A docking study of the most active compounds **26**, **34** and **35** into *Candida albicans* lanosterol 14α-demethylase reveals that these compounds have more favorable estimated binding free energy (−9.9 to −9.1 kcal/mol) than the reference drug fluconazole (−8.1 kcal/mol). The most probable binding model obtained for the tested compounds shows that the substituent from position 4 of the pyridine ring is involved in an interaction with heme cofactor and binding site residues rather than the 5-amino-1*H*-1,2,4-triazole moiety which is oriented in the opposite direction into the hydrophobic access tunnel. This finding suggests that it would be reasonable for perform further modifications to focus on enlargement of the triazole fragment to better fit into to the hydrophobic tunnel, and on the optimization of the pyridine 4 substituent.

## Figures and Tables

**Table 1:** The MIC values (µg/mL) obtained for compounds **26**–**36** and fluconazole (**Fl**) against yeast strains used in assay.

Compd.	M.W:	*Candida albicans (9 strains)*	*Candida glabrata (5 strains)*	*Candida guilliermondii (2 strains)*	*Candida krusei (4 strains)*	*Candida lusitaniae (3 strains)*	*Candida parapsilosis (4 strains)*	*Candida tropicalis (4 strains)*	*Candida utilis (1 strain)*	*Geotrichum candidum (2 strains)*	*Rhodotorula mucilaginosa (2 strains)*	*Saccharomyces cerevisiae (1 strain)*
**26**	400.46	≤6.2; 50; 100; 100; 100; *; *; *; * ^A^	*; *; *; *; 100 ^B^	12.5; 25	100; *; *; 100 ^C^	100; *; 12.5 ^D^	*; *; *; *, ^E^	100; *; *; 100 ^F^	*	*; *	≤6.2; 50	*
**27**	434.90	*; *; *; *;*; *; *; *; * ^A^	*; *; *; *; * ^B^	*; *	*; *; *; * ^C^	*; *; *, ^D^	*; *; *; *, ^E^	*; *; *; *, ^F^	*	*; *	*; *	*
**28**	418.45	12.5; 12.5; 100; 100; *; *; *; *;* ^A^	*; *; *; *; * ^B^	50; 100	100; *; *; * ^C^	50; 100; 50 ^D^	*; *; *; * ^E^	*; *; *; * ^F^	100	*; *	12.5; 25	*
**29**	469.35	*; *; *; *;*; *; *; *; * ^A^	*; *; *; *; * ^B^	*; *	*; *; *; * ^C^	*; *; * ^D^	*; *; *; * ^E^	*; *; *; * ^F^	*	*; *	*; *	*
**30**	430.48	12.5; 100; 100; *; *; *; *;*; * ^A^	*; *; *; *;* ^B^	*; *	*; *; *; * ^C^	*; *;* ^D^	*; *; *; * ^E^	*; *; *; * ^F^	*	*; *	100; *	*
**31**	334.36	25; 50; *; *; *; *;*; *; * ^A^	*; *; *; *; * ^B^	*; *	*; *; *; * ^C^	*; *; * ^D^	*; *; *; * ^E^	25; *; *; 50 ^F^	*	*; *	100; *	*
**32**	348.38	25; 50; 100; *; *; *; *;*; * ^A^	50; *; *; * ^B^	*; *	25; *; *; 50 ^C^	*; *; * ^D^	*; *; *; * ^E^	100 *; *; 100 ^F^	100	*; *	25; 25	*
**33**	390.46	*; *; *; *;*; *; *; *; * ^A^	*; *; *; *; * ^B^	*; *	*; *; *; * ^C^	*; *; * ^D^	*; *; *; * ^E^	*; *; *; * ^F^	*	*; *	*; *	*
**34**	362.41	≤6.2; ≤6.2; 100; *; *; *; *;*; * ^A^	*; *; *; *; * ^B^	*; *	100; *; *;* ^C^	50; 100; 100 ^D^	*; *; *; * ^E^	*; *; *; * ^F^	*	*; *	100; 200	*
**35**	362.43	≤6.2; 12.5; 50; 100; 100; *; *; *; 50 ^A^	100; 100; *; *; 100 ^B^	100; *	12.5;*; *; 50 ^C^	*; *; * ^D^	*; *; *; * ^E^	*; *; *; * ^F^	*	*; *	6.2; 100	*
**36**	329.36	12.5; 50; 100; 100; *; *; *; *; 100 ^A^	25; 100; *; *; 100 ^B^	50; 100	100; *; *; 100 ^C^	100; *; 100 ^D^	*; *; *; * ^E^	*; *; *; * ^F^	*	*; *	25; 100	*
**Fl**	306.28	12.5; 50; #; #; #; #; #; #; # ^A^	25; 50; #; #; 25 ^B^	12.5; 50	25; 50; 50; 50 ^C^	12.5; 12.5; 12.5 ^D^	6.2; 12.5; 25; 3.1 ^E^	25; 50; #; 6.2 ^F^	25	12.5; 25	#; #	#

M.W.—molecular weight; * ≥200 (µg/mL); # >100 (µg/mL); ^A^—standard strain *Candida albicans* ATCC 10231; ^B^—standard strain *Candida glabrata* ATCC 66032; ^C^—standard strain *Candida krusei* ATCC 14243; ^D^—standard strain *Candida lusitaniae* ATCC 34499; ^E^—standard strain *Candida parapsilosis* ATCC 2201; ^F^—standard strain *Candida tropicalis* ATCC 750.

**Table 2:** Predicted binding affinity and non-covalent interaction between compounds **26**, 3**4** and **35** and the active site of CYP51.

Compound Binding Affinity	Molecule Fragment Involved in Interaction	Interacting Residue	Type of Interaction	Interaction Distance [Å]
**26** −9.9 (kcal/mol)	5-amino-1*H*-1,2,4-triazole	LEU 376	π-alkyl	4.93
		SER378	hydrogen bond	2.67
		PHE380	π-donor hydrogen	4.93
		MET508	π-sulfur	2.67
	pyridine-3-sulfonamide scaffold	PHE228	π-π	5.13
		GLY307	π-donor hydrogen	3.56
	4-phenylpiperazine substituent	TYR132	π-alkyl	5.24
		HEME	π-alkyl	5.22
		HEME	π-σ	3.23
		ILE304	π-alkyl	5.42
		ILE131	π-σ	3.70
		ILE131	π-σ	3.51
**34** −9.3 (kcal/mol)	5-amino-1*H*-1,2,4-triazole	LEU 376		5.06
		SER378	hydrogen bond	2.75 (OH) 2.77 (C=O)
		PHE233	π-π	2.29
	pyridine-3-sulfonamide scaffold	PHE228	π-π	5.05
		LEU 376	π-alkyl	5.41
		MET508	π-alkyl	5.38
	3,5-diethyl-1*H*-pyrazole substituent	LEU 376	π-alkyl	4.03
		ILE131	π-alkyl	4.66
		HEME	π-σ	3.69
		HEME	π-π	4.97
		HEME	π-alkyl	3.71
**35** −9.1 (kcal/mol)	5-amino-1*H*-1,2,4-triazole	LEU 376	π-alkyl	4.39
		SER378	hydrogen bond	1.83
		TYR118	hydrogen bond	2.19
	pyridine-3-sulfonamide scaffold	LEU 376	π-alkyl	5.27
		PHE228	π-π	5.66
	benzylthio substituent	ILE131	π-alkyl	5.16
		HEME	π-σ	4.18
		HEME	π-σ	3.74

## Supplementary Materials

Supplementary materials can be accessed online, Figure S1: Synthesis of 4-substituted pyridine-3-sulfonamide substrates **2**–**3**, **8**–**11** and **12**–**14**, Table S1: Inhibition growth percent (IGP [%]) of compounds **20**, **26**, **28**–**31** and **34**–**36** against selected (IGP ≥10) NCI-60 cancer cell lines at single concentration of 10^−5^ M., ^1^H-NMR spectrum of compounds **17**, **21**, **25**, **28**, **32** and **36**.

## References

[1] Swain S.S., Paidesetty S.K., Padhy R.N. (2017). Antibacterial activity, computational analysis and host toxicity study of thymol-sulfonamide conjugates. Biomed. Pharmacother..

[2] Capasso C., Supuran C.T. (2013). Anti-infective carbonic anhydrase inhibitors: A patent and literature review. Expert Opin. Ther. Pat..

[3] Rendell M. (2004). The role of sulphonylureas in the management of type 2 diabetes mellitus. Drugs.

[4] Supuran C.T. (2008). Diuretics: From classical carbonic anhydrase inhibitors to novel applications of the sulfonamides. Curr. Pharm. Des..

[5] Kaur I.P., Smitha R., Aggarwal D., Kapil M. (2002). Acetazolamide: Future perspective in topical glaucoma therapeutics. Int. J. Pharm..

[6] Angius F., Piras E., Uda S., Madeddu C., Serpe R., Bigi R., Chen W., Dittmer D.P., Pompei R., Ingianni A. (2017). Antimicrobial sulfonamides clear latent Kaposi sarcoma herpesvirus infection and impair MDM2 p53 complex formation. J. Antibiot. (Tokyo).

[7] Singh A., Yadav M., Srivastava R., Singh N., Kaur R., Gupta S.K., Singh R.K. (2016). Design and anti-HIV activity of arylsulphonamides as non-nucleoside reverse transcriptase inhibitors. Med. Chem. Res..

[8] Barone M., Pannuzzo G., Santagati A., Catalfo A., De Guidi G., Cardile V. (2014). Molecular docking and fluorescence characterization of benzothieno[3,2-*d*]pyrimidin-4-one sulphonamide thio-derivatives, a novel class of selective cyclooxygenase-2 Inhibitors. Molecules.

[9] Nissinen L., Ojala M., Langen B., Dost R., Pihlavisto M., Käpylä J., Marjamäki A., Heino J. (2015). Sulfonamide inhibitors of α2β1 integrin reveal the essential role of collagen receptors in in vivo models of inflammation. Pharmacol. Res. Perspect..

[10] Uehara T., Minoshima Y., Sagane K., Sugi N.H., Mitsuhashi K.O., Yamamoto N., Kamiyama H., Takahashi K., Kotake Y., Uesugi M. (2017). Selective degradation of splicing factor CAPERα by anticancer sulfonamides. Nat. Chem. Biol..

[11] Supuran C.T. (2017). Carbonic Anhydrase Inhibition and the Management of Hypoxic Tumors. Metabolites.

[12] Żołnowska B., Sławiński J., Pogorzelska A., Szafrański K., Kawiak A., Stasiłojć G., Belka M., Zielińska J., Bączek T. (2017). Synthesis, QSAR studies, and metabolic stability of novel 2-alkylthio-4-chloro-*N*-(5-oxo-4,5-dihydro-1,2,4-triazin-3-yl)benzenesulfonamide derivatives as potential anticancer and apoptosis-inducing agents. Chem. Biol. Drug Des..

[13] Brzozowski Z., Sławiński J., Sączewski F., Innocenti A., Supuran C.T. (2010). Carbonic anhydrase inhibitors: Synthesis and inhibition of the human cytosolic isozymes I and II and transmembrane isozymes IX, XII (cancer-associated) and XIV with 4-substituted 3-pyridinesulfonamides. Eur. J. Med. Chem..

[14] Brzozowski Z., Sławiński J., Innocenti A., Supuran C.T. (2010). Carbonic anhydrase inhibitors. Regioselective synthesis of novel 1-substituted 1,4-dihydro-4-oxo-3-pyridinesulfonamides and their inhibition of the human cytosolic isozymes I and II and transmembrane cancer-associated isozymes IX and XII. Eur. J. Med. Chem..

[15] Sławiński J., Szafrański K., Vullo D., Supuran C.T. (2013). Carbonic anhydrase inhibitors. Synthesis of heterocyclic 4-substituted pyridine-3-sulfonamide derivatives and their inhibition of the human cytosolic isozymes I and II and transmembrane tumor-associated isozymes IX and XII. Eur. J. Med. Chem..

[16] Szafrański K., Sławiński J. (2015). Synthesis of Novel 1-(4-Substituted pyridine-3-sulfonyl)-3-phenylureas with Potential Anticancer Activity. Molecules.

[17] Perfect J.R. (2016). “Is there an emerging need for new antifungals?”. Expert Opin. Emerg. Drugs.

[18] Richardson M.D. (2005). Changing patterns and trends in systemic fungal infections. J. Antimicrob. Chemother..

[19] Strushkevich N., Usanov S.A., Park H.W. (2010). Structural basis of human CYP51 inhibition by antifungal azoles. J. Mol. Biol..

[20] Sheehan D.J., Hitchcock C.A., Carol M. (1999). Current and Emerging Azole Antifungal Agents. Clin. Microbiol. Rev..

[21] Kharb R., Sharma P.C., Yar M.S. (2011). Pharmacological significance of triazole scaffold. J. Enzym. Inhib. Med. Chem..

[22] Li X., Liu C., Tang S., Wu Q., Hu H., Zhao Q., Zou Y. (2016). Synthesis, In Vitro Biological Evaluation, and Molecular Docking of New Triazoles as Potent Antifungal Agents. Arch. Pharm..

[23] Ahmed S., Zayed M., El-Messery S., Al-Agamy M., Abdel-Rahman H. (2016). Design, Synthesis, Antimicrobial Evaluation and Molecular Modeling Study of 1,2,4-Triazole-Based 4-Thiazolidinones. Molecules.

[24] Pandey S.K., Ahamd A., Pandey O.P., Nizamuddin K. (2014). Polyethylene Glycol Mediated, One-Pot, Three-Component Synthetic Protocol for Novel 3-[3-Substituted-5-mercapto-1,2,4-triazol-4-yl]-spiro-(indan-1′,2-thiazolidin)-4-ones as New Class of Potential Antimicrobial and Antitubercular Agents. J. Heterocycl. Chem..

[25] Zoumpoulakis P., Camoutsis C., Pairas G., Soković M., Glamočlija J., Potamitis C., Pitsas A. (2012). Synthesis of novel sulfonamide-1,2,4-triazoles, 1,3,4-thiadiazoles and 1,3,4-oxadiazoles, as potential antibacterial and antifungal agents. Biological evaluation and conformational analysis studies. Bioorg. Med. Chem..

[26] Somagond S.M., Kamble R.R., Kattimani P.P., Joshi S.D., Dixit S.R. (2017). Design, synthesis, docking and in vitro antifungal study of 1,2,4-triazole hybrids of 2-(aryloxy)quinolines. Heterocycl. Commun..

[27] Ozdemir S.B., Demirbas N., Cebeci Y.U., Bayrak H., Mermer A., Ceylan S., Demirbas A. (2017). Synthesis and Antimicrobial Activities of Hybrid Heterocyclic Molecules Based on 1-(4-Fluorophenyl)piperazine Skeleton. Lett. Drug Des. Discov..

[28] Karaca Gençer H., Acar Çevik U., Levent S., Sağlık B.N., Korkut B., Özkay Y., Ilgın S., Öztürk Y. (2017). New Benzimidazole-1,2,4-Triazole Hybrid Compounds: Synthesis, Anticandidal Activity and Cytotoxicity Evaluation. Molecules.

[29] Mansueto R., Perna F.M., Salomone A., Perrone S., Florio S., Capriati V. (2014). Efficient Regioselective Synthesis of 3,4,5-Trisubstituted 1,2,4-Triazoles on the Basis of a Lithiation-Trapping Sequence. European J. Org. Chem..

[30] Katritzky A.R., Tymoshenko D.O., Chen K., Fattah A.A. (2001). Ring and side chain reactivities of 1-([1,3,4]oxadiazol-2-ylmethyl)-1*H*-benzotriazoles. ARKIVOC.

[31] Kleschick W.A., Dunbar J.E., Snider S.W., Vinogradoff A. (1988). Regiospecific Synthesis of Arenesulfonamide Derivatives of 3,5-Diamino-1,2,4-triazole. J. Org. Chem..

[32] Wouters J., Michaux C., Durant F., Michel Dogné J., Delarge J., Masereel B. (2000). Isosterism among analogues of torasemide: Conformational, electronic and lipophilic properties. Eur. J. Med. Chem..

[33] Hargrove T.Y., Friggeri L., Wawrzak Z., Qi A., Hoekstra W.J., Schotzinger R.J., York J.D., Peter Guengerich F., Lepesheva G.I. (2017). Structural analyses of *Candida albicans* sterol 14α-demethylase complexed with azole drugs address the molecular basis of azole-mediated inhibition of fungal sterol biosynthesis. J. Biol. Chem..

[34] Trott O., Olson A.J. (2010). AutoDock Vina: improving the speed and accuracy of docking with a new scoring function, efficient optimization, and multithreading. J. Comput. Chem..

[35] Chen C.K., Leung S.S.F., Guilbert C., Jacobson M.P., Mckerrow J.H., Podust L.M. (2010). Structural characterization of CYP51 from *Trypanosoma cruzi* and *Trypanosoma brucei* bound to the antifungal drugs posaconazole and fluconazole. PLoS Negl. Trop. Dis..

[36] Forbes B., Sahm D., Weissfeld A. (2007). Bailey & Scott’s Diagnostic Microbiology.

[37] Winn W., Allen S., Janda W., Koneman E., Procop G., Schrekenberger P., Woods G. (2006). Koneman’s Color Atlas and Textbook of Diagnostic Microbiology.

[38] Morris G.M., Huey R., Lindstrom W., Sanner M.F., Belew R.K., Goodsell D.S., Olson A.J. (2009). AutoDock4 and AutoDockTools4: Automated docking with selective receptor flexibility. J. Comput. Chem..

[39] Shoemaker R.H. (2006). The NCI60 human tumour cell line anticancer drug screen. Nat. Rev. Cancer.

[40] Boyd M.R., Paull K.D. (1995). Some practical considerations and applications of the national cancer institute in vitro anticancer drug discovery screen. Drug Dev. Res..

