# Supplementary material for: Syntheses of Novel 4-Substituted N-(5-amino-1H-1,2,4-triazol-3-yl)pyridine-3-sulfonamide Derivatives with Potential Antifungal Activity
Source: Molecules. 2017 Nov 7;22(11):1926. doi: 10.3390/molecules22111926 (PMC6150321; doi:10.3390/molecules22111926)

Supplementary Materials

Syntheses of Novel 4-Substituted *N*-(5-amino-1*H*-1,2,4-triazol-3-yl) pyridine-3-sulfonamide Derivatives with Potential Antifungal Activity

Krzysztof Szafrański [1]*, Jarosław Sławiński [1]*, Anna Kędzia[2] and Ewa Kwapisz [2,]

[1] Department of Organic Chemistry, Medical University of Gdańsk, Al. Gen. J. Hallera 107., 80-416 Gdańsk, Poland; e-mails: k.szafranski@gumed.edu.pl (K.S.), jaroslaw@gumed.edu.pl (J.S.)

[2] Department of Oral Microbiology, Medical University of Gdańsk, ul. Dębowa 25., 80-204, Gdańsk, Poland; e-mails: anak@gumed.edu.pl (A.K.), kwapisz@gumed.edu.pl (E.K.)

* Correspondence: k.szafranski@gumed.edu.pl (K.S.), jaroslaw@gumed.edu.pl (J.S.); Tel.: +48-58-349-1098; Fax: +48-58-349-1277

Table of Contents

Scheme S1. 2

Table S1.. 3

Spectrum 1. ^{1}H-NMR of compound **17** (500 MHz, DMSO-d_6). 4

Spectrum 2. ^{1}H-NMR of compound **21** (500 MHz, DMSO-d_6). 5

Spectrum 3. ^{1}H-NMR of compound **25** (500 MHz, DMSO-d_6). 6

Spectrum 4. ^{1}H-NMR of compound **28** (500 MHz, DMSO-d_6). 7

Spectrum 5. ^{1}H-NMR of compound **32** (200 MHz, DMSO-d_6). 8

Spectrum 6. ^{1}H-NMR of compound **36** (500 MHz, DMSO-d_6). 9

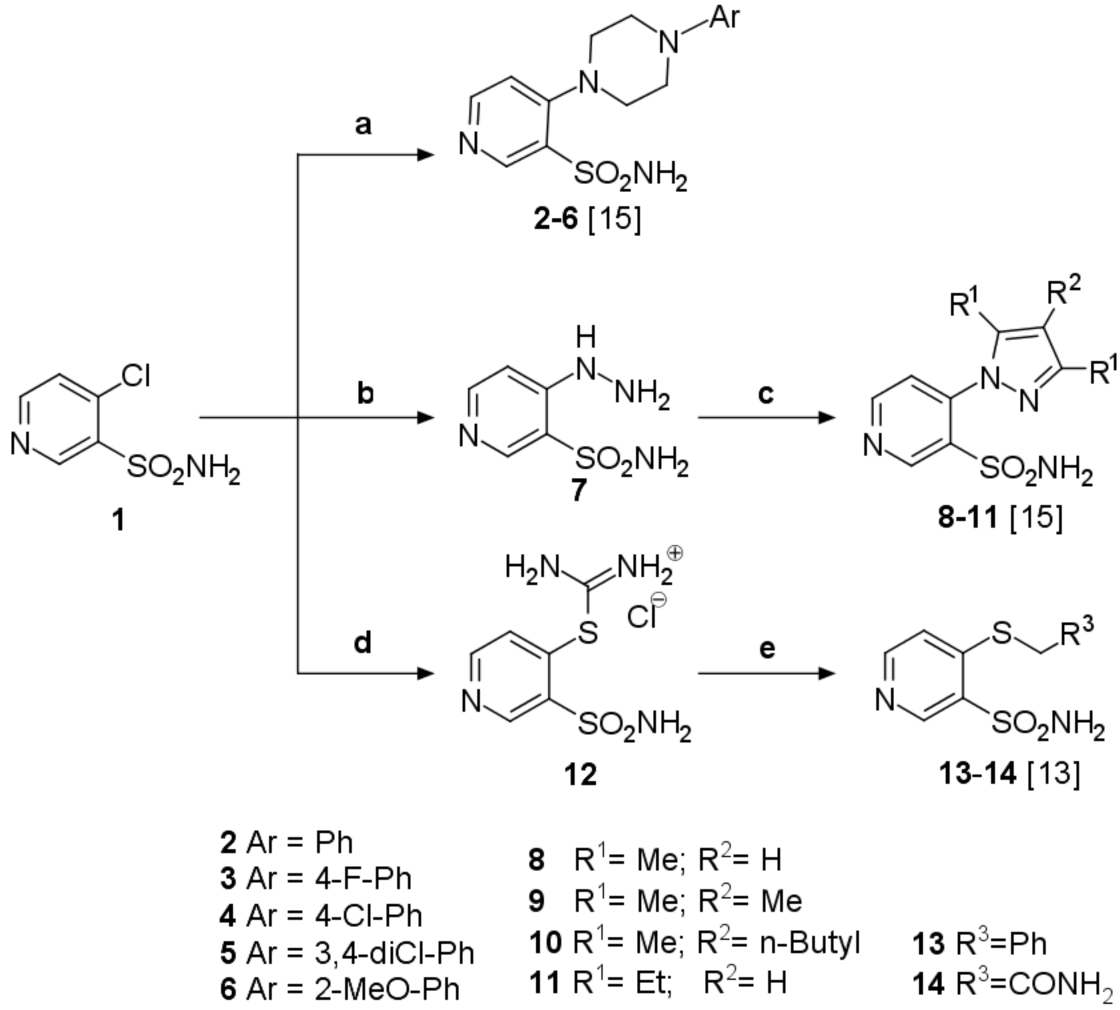

Reagents and conditions:
(**a**) 4-Ar-piperazine (2 eq.), MeOH, r.t. 28 h, or 4-Ar-piperazine hydrochloride (1.2 eq.), Et_3N (2.25 eq.), MeOH r.t. 72 h; (**b**) H_2N-NH_2xH_2O (6 eq.), MeOH r.t. 16 h, reflux, 4 h; (**c**)1,3-diketone, AcOH, reflux, 2 h; (**d**) thiourea (1.02 eq.), MeCN, reflux, 3 h; (**e**) NaOH (2.14 eq.) water/THF 5 °C, 0.5 h, r.t. 5 h.

Scheme S1. Synthesis of 4-substituted pyridine-3-sulfonamide substrates **2-3**, **8-11** **[**15**]** and **12-14** [13] .

[13] Brzozowski, Z.; Sławiński, J.; Sączewski, F.; Innocenti, A.; Supuran, C. T. Carbonic anhydrase inhibitors: Synthesis and inhibition of the human cytosolic isozymes I and II and transmembrane isozymes IX, XII (cancer-associated) and XIV with 4-substituted 3-pyridinesulfonamides. *Eur. J. Med. Chem.* **2010**, *45*, 2396–2404, doi:10.1016/j.ejmech.2010.02.020.

[15] Sławiński, J.; Szafrański, K.; Vullo, D.; Supuran, C. T. Carbonic anhydrase inhibitors. Synthesis of heterocyclic 4-substituted pyridine-3-sulfonamide derivatives and their inhibition of the human cytosolic isozymes I and II and transmembrane tumor-associated isozymes IX and XII. *Eur. J. Med. Chem.* **2013**, *69*, 701–10, doi:10.1016/j.ejmech.2013.09.027.

Table S1. Inhibition growth percent (IGP [%]) of compounds **20**, **26**, **28**–**31** and **34**–**36** against selected (IGP ≥10) NCI-60 cancer cell lines at single concentration of 10^{-5} M.

	IGP [%]								
Panel/cell line	**20**	**26**	**28**	**29**	**30**	**31**	**34**	**35**	**36**
Leukemia									
MOLT-4	*	9	*	*	*	13	7	1	*
SR	NT	*	3	*	NT	7	6	13	NT
Non-small-cell lung cancer									
HOP-92	13	*	NT	13	6	2	2	NT	NT
NCI-H322M	*	3	*	12	8	*	*	*	*
Colon cancer									
HCC-2998	NT	13	*	5	*	2	18	*	*
CNS cancer									
SF-539	*	*	1	NT	*	*	*	3	10
SNB-75	NT	9	*	4	4	7	1	*	21
Melanoma									
MALME-3M	*	1	1	2	17	11	6	*	*
Renal cancer									
A498	*	*	*	*	18	*	*	*	7
CAKI-1	13	1	*	13	*	*	2	*	6
TK-10	NT	NT	*	*	12	NT	*	*	*
UO-31	8	17	10	16	16	*	5	9	5
Breast cancer									
MCF7	*	*	*	*	*	1	23	*	2
T-47D	*	3	7	8	12	1	6	1	7

Spectrum 1. ^{1}H-NMR of compound **17** (500 MHz, DMSO-d_6).

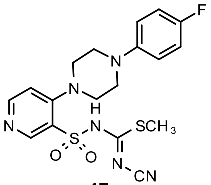

17

INDEX	FREQUENCY	PPM	HEIGHT
1	4401.459	8.806	38.5
2	4149.490	8.302	19.4
3	4142.166	8.287	20.3
4	3679.735	7.362	27.5
5	3672.410	7.347	27.2
6	3539.590	7.082	14.4
7	3530.312	7.063	34.7
8	3522.011	7.047	24.4
9	3501.502	7.005	27.3
10	3496.618	6.996	30.4
11	3492.224	6.987	21.2
12	3487.340	6.977	17.6
13	2020.942	4.043	55.0
14	2016.059	4.034	43.7
15	1676.195	3.354	3.8
16	1633.223	3.268	43.9
17	1628.829	3.259	58.4
18	1623.946	3.249	44.1
19	1244.040	2.489	34.8
20	1174.699	2.350	138.0

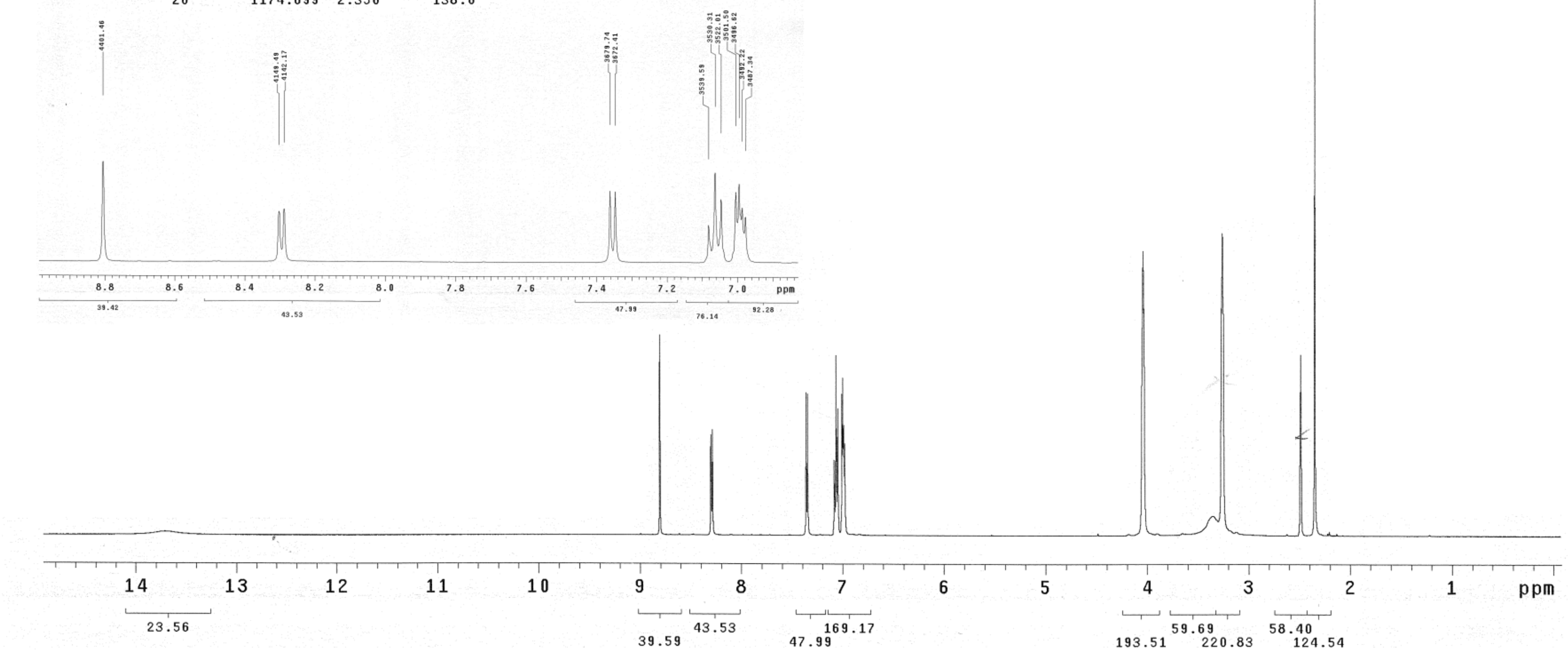

Spectrum 2. ^{1}H-NMR of compound **21** (500 MHz, DMSO-d_6).

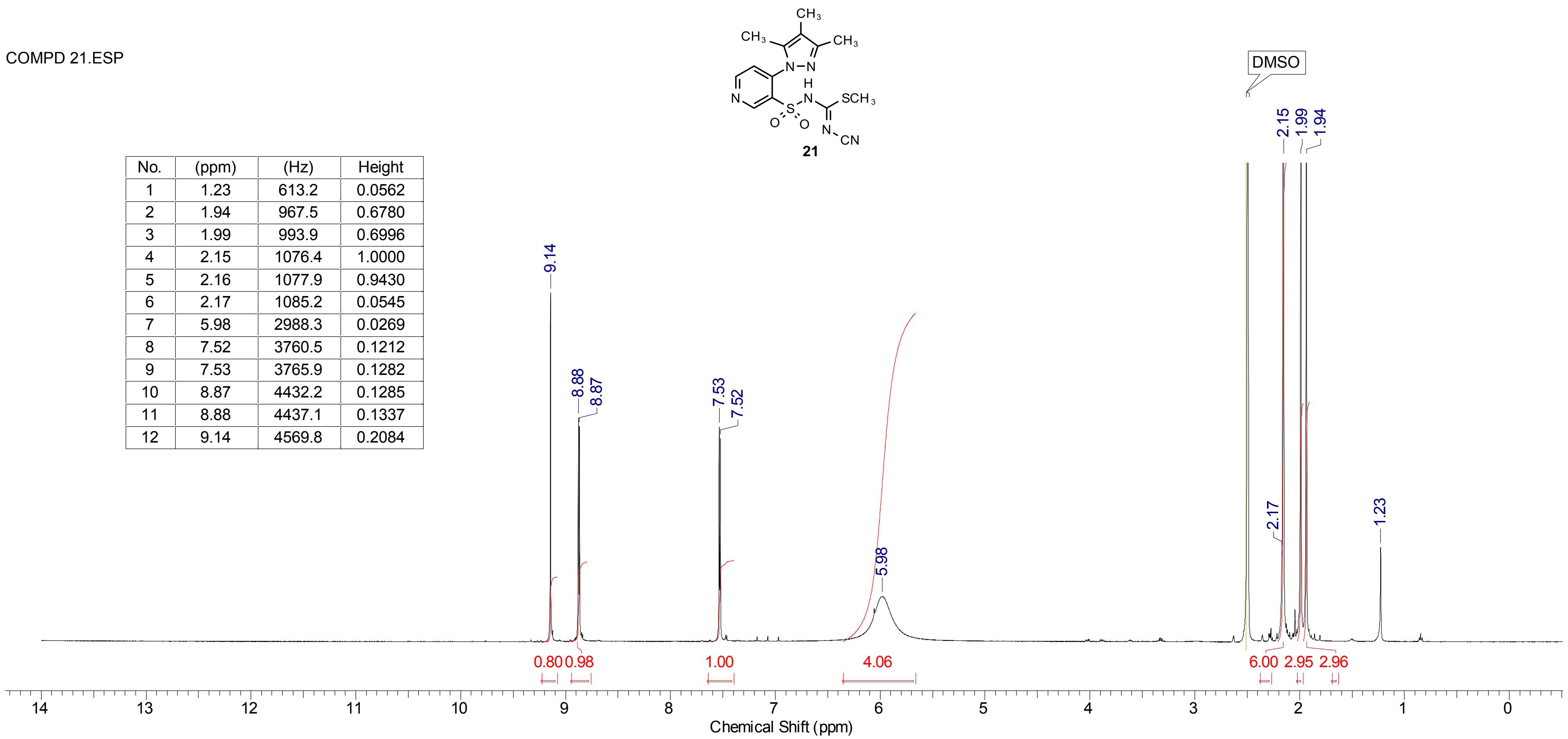

No.	(ppm)	(Hz)	Height
1	1.23	613.2	0.0562
2	1.94	967.5	0.6780
3	1.99	993.9	0.6996
4	2.15	1076.4	1.0000
5	2.16	1077.9	0.9430
6	2.17	1085.2	0.0545
7	5.98	2988.3	0.0269
8	7.52	3760.5	0.1212
9	7.53	3765.9	0.1282
10	8.87	4432.2	0.1285
11	8.88	4437.1	0.1337
12	9.14	4569.8	0.2084

Spectrum 3. ^{1}H-NMR of compound **25** (500 MHz, DMSO-d_6).

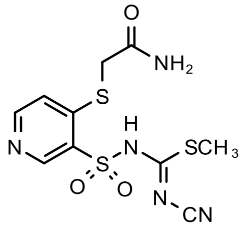

25

INDEX	FREQUENCY	PPM	HEIGHT
1	4427.828	8.859	44.6
2	4315.516	8.634	24.0
3	4309.168	8.621	24.1
4	3906.800	7.816	26.5
5	3900.452	7.804	25.3
6	3860.410	7.724	19.5
7	3682.665	7.368	18.5
8	1981.878	3.965	101.0
9	1244.040	2.489	31.4
10	1181.047	2.363	186.1

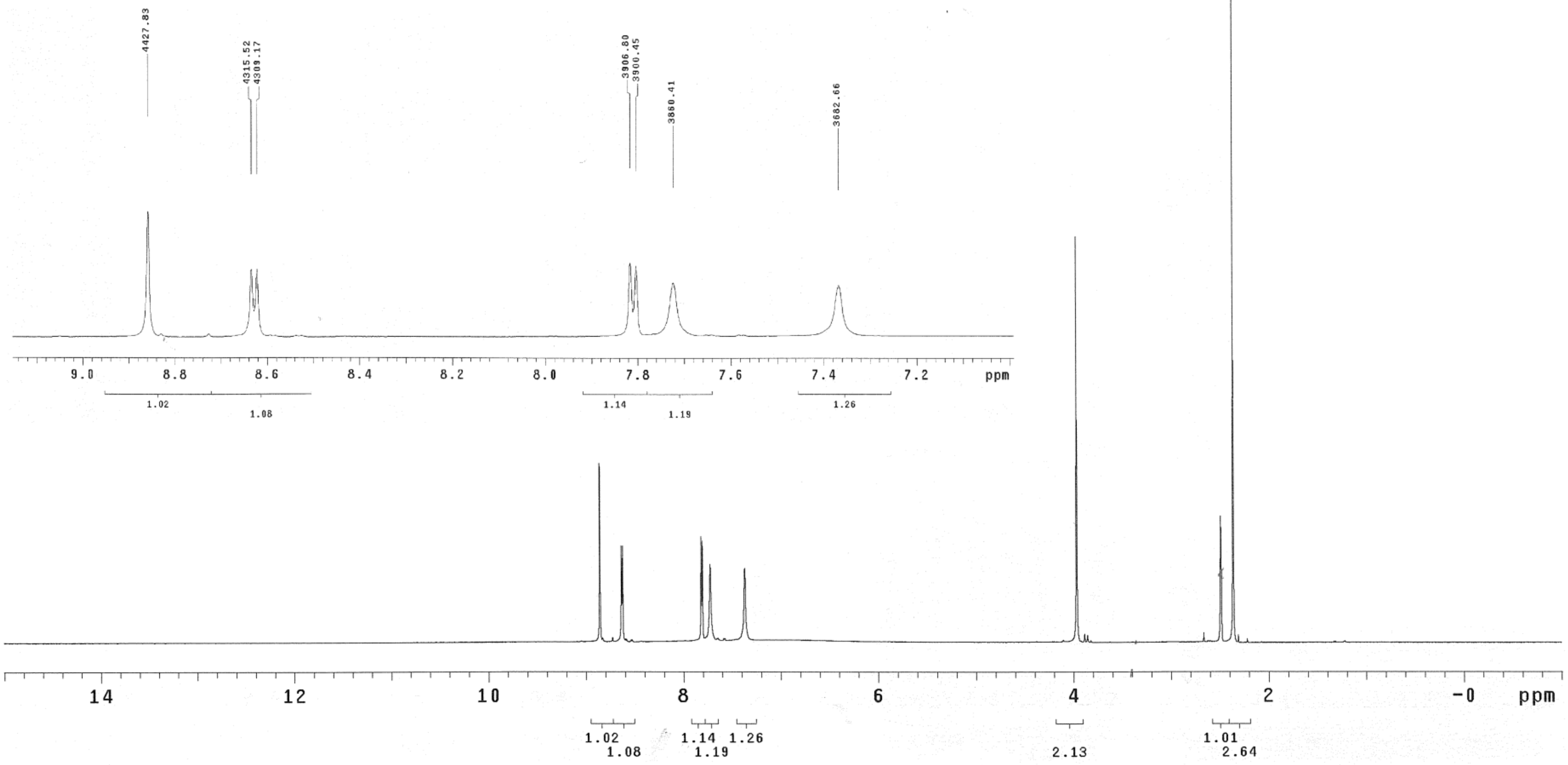

Spectrum 4. ^{1}H-NMR of compound **28** (500 MHz, DMSO-d_6).

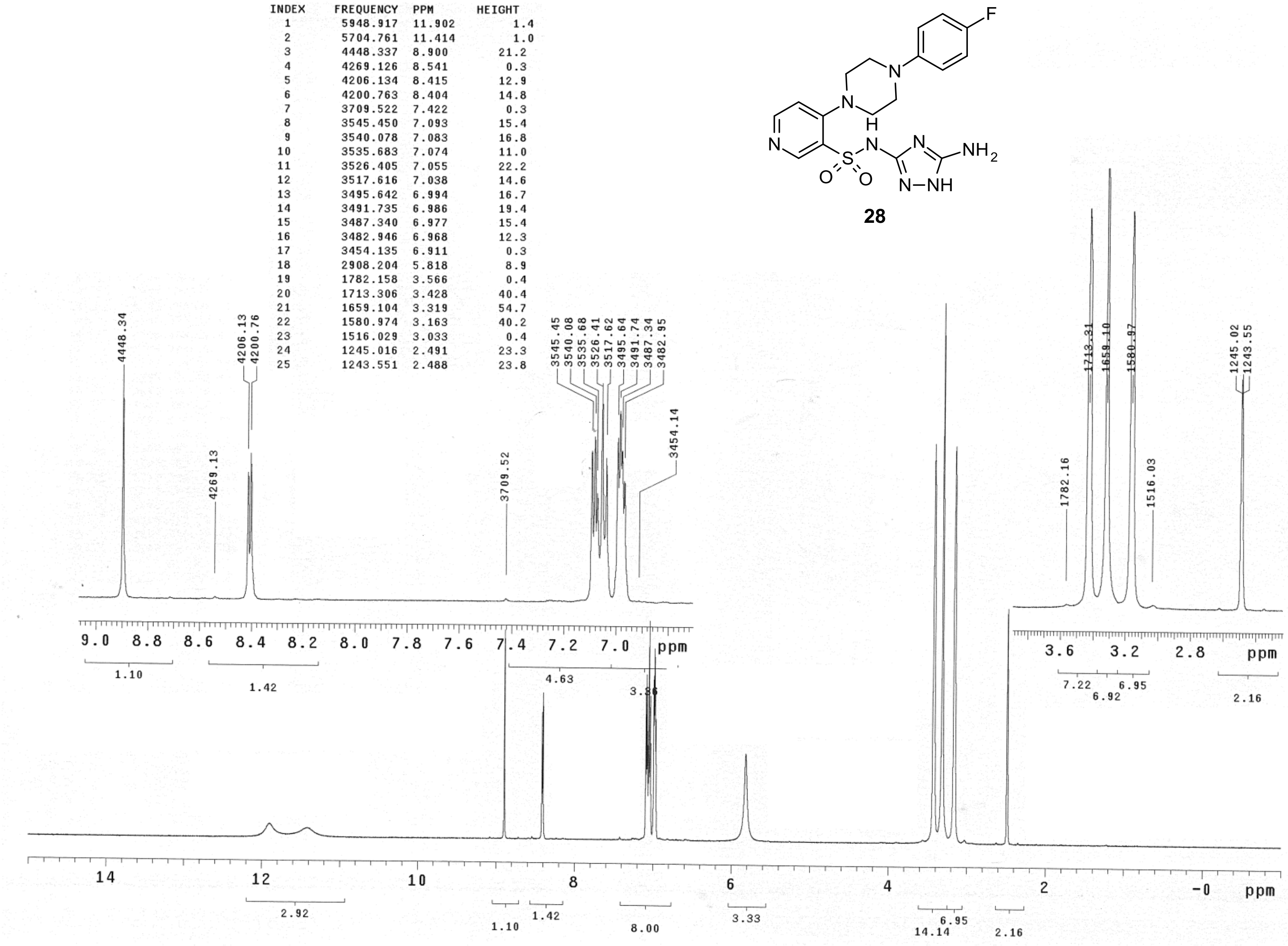

INDEX	FREQUENCY	PPM	HEIGHT
1	5948.917	11.902	1.4
2	5704.761	11.414	1.0
3	4448.337	8.900	21.2
4	4269.126	8.541	0.3
5	4206.134	8.415	12.9
6	4200.763	8.404	14.8
7	3709.522	7.422	0.3
8	3545.450	7.093	15.4
9	3540.078	7.083	16.8
10	3535.683	7.074	11.0
11	3526.405	7.055	22.2
12	3517.616	7.038	14.6
13	3495.642	6.994	16.7
14	3491.735	6.986	19.4
15	3487.340	6.977	15.4
16	3482.946	6.968	12.3
17	3454.135	6.911	0.3
18	2908.204	5.818	8.9
19	1782.158	3.566	0.4
20	1713.306	3.428	40.4
21	1659.104	3.319	54.7
22	1580.974	3.163	40.2
23	1516.029	3.033	0.4
24	1245.016	2.491	23.3
25	1243.551	2.488	23.8

Spectrum 5. ^{1}H-NMR of compound **32** (200 MHz, DMSO-d_6).

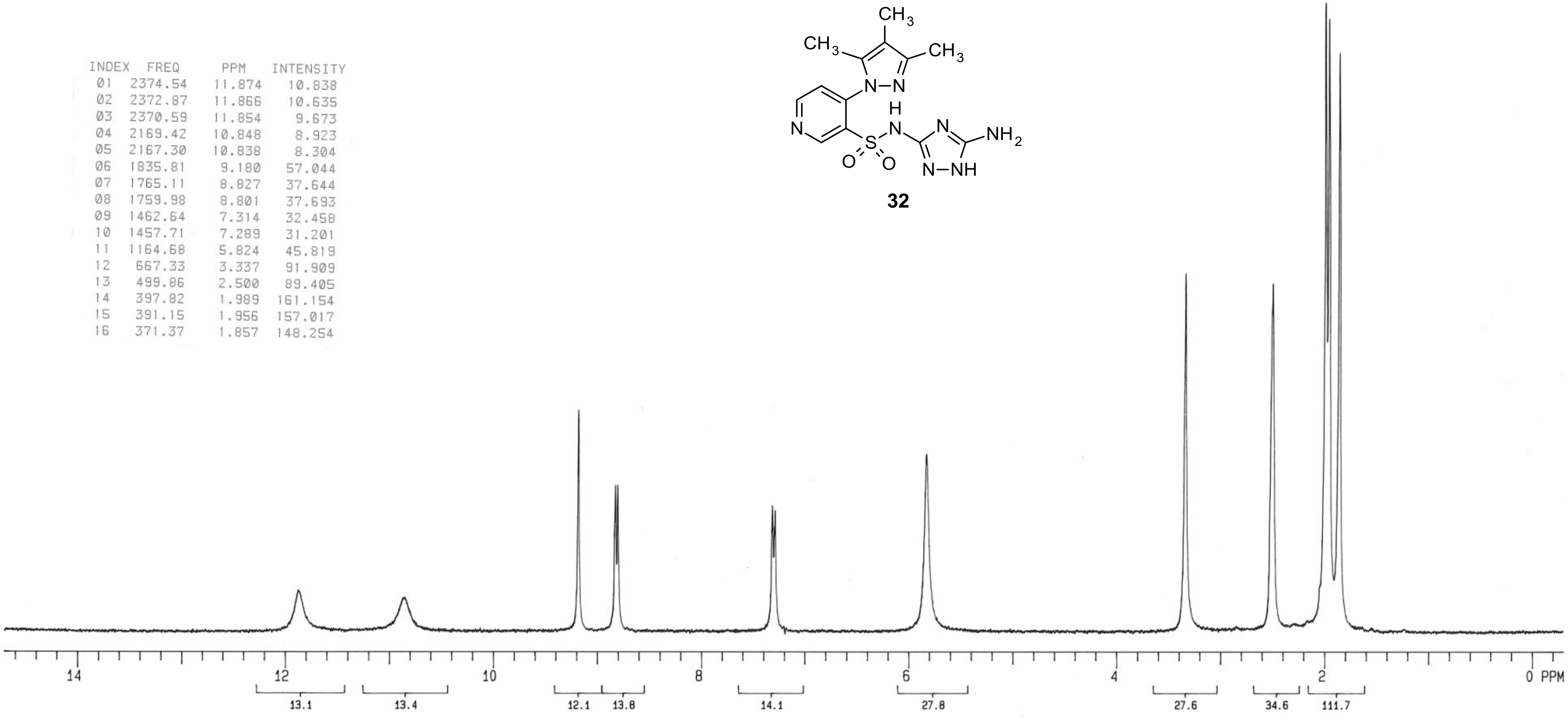

INDEX	FREQ	PPM	INTENSITY
01	2374.54	11.874	10.838
02	2372.87	11.866	10.635
03	2370.59	11.854	9.673
04	2169.42	10.848	8.923
05	2167.30	10.838	8.304
06	1835.81	9.180	57.044
07	1765.11	8.827	37.644
08	1759.98	8.801	37.693
09	1462.64	7.314	32.458
10	1457.71	7.289	31.201
11	1164.68	5.824	45.819
12	667.33	3.337	91.909
13	499.86	2.500	89.405
14	397.82	1.989	161.154
15	391.15	1.956	157.017
16	371.37	1.857	148.254

Spectrum 6. ^{1}H-NMR of compound **36** (500 MHz, DMSO-d_6).

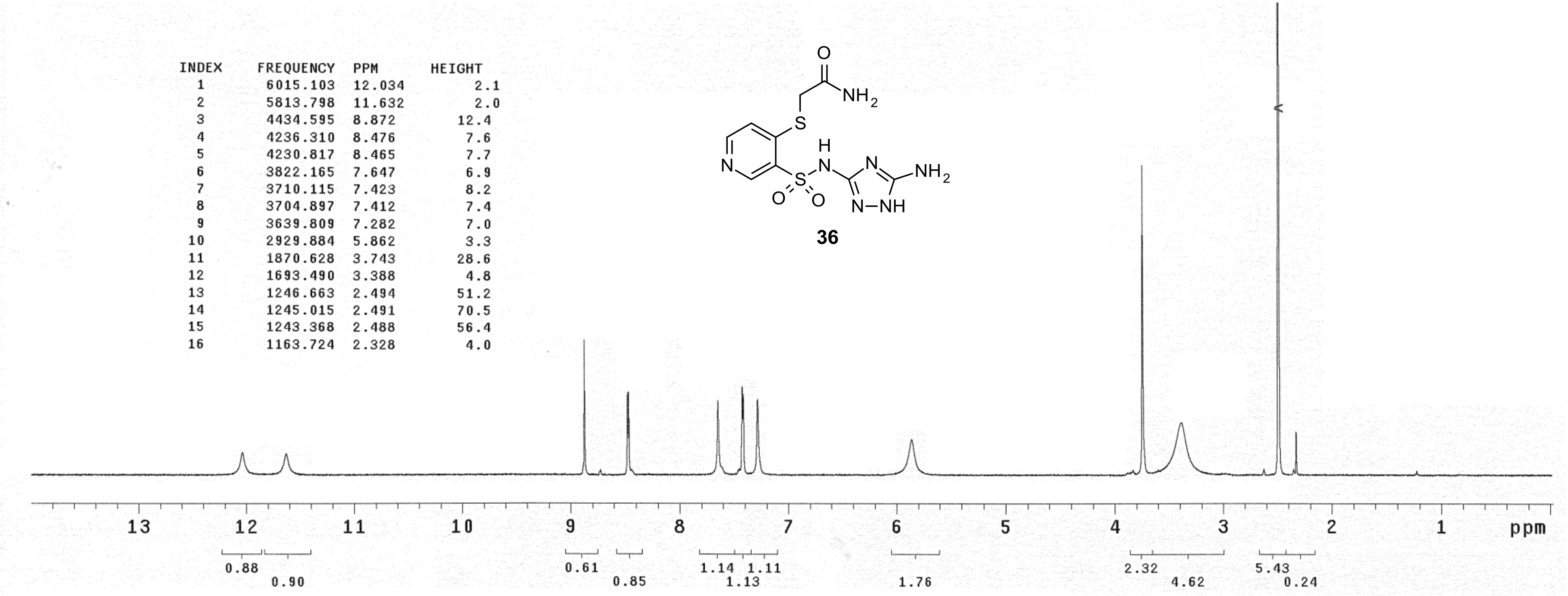

Supplement: Supplementary file 1 [file molecules-22-01926-s001.pdf]
